# The Effectiveness of Blended Learning in Health Professions: Systematic Review and Meta-Analysis

**DOI:** 10.2196/jmir.4807

**Published:** 2016-01-04

**Authors:** Qian Liu, Weijun Peng, Fan Zhang, Rong Hu, Yingxue Li, Weirong Yan

**Affiliations:** ^1^ Department of Epidemiology and Biostatistics School of Public Health Tongji Medical College of Huazhong University of Science &Technology Wuhan China

**Keywords:** blended learning, effectiveness, knowledge, health professions, meta-analysis

## Abstract

**Background:**

Blended learning, defined as the combination of traditional face-to-face learning and asynchronous or synchronous e-learning, has grown rapidly and is now widely used in education. Concerns about the effectiveness of blended learning have led to an increasing number of studies on this topic. However, there has yet to be a quantitative synthesis evaluating the effectiveness of blended learning on knowledge acquisition in health professions.

**Objective:**

We aimed to assess the effectiveness of blended learning for health professional learners compared with no intervention and with nonblended learning. We also aimed to explore factors that could explain differences in learning effects across study designs, participants, country socioeconomic status, intervention durations, randomization, and quality score for each of these questions.

**Methods:**

We conducted a search of citations in Medline, CINAHL, Science Direct, Ovid Embase, Web of Science, CENTRAL, and ERIC through September 2014. Studies in any language that compared blended learning with no intervention or nonblended learning among health professional learners and assessed knowledge acquisition were included. Two reviewers independently evaluated study quality and abstracted information including characteristics of learners and intervention (study design, exercises, interactivity, peer discussion, and outcome assessment).

**Results:**

We identified 56 eligible articles. Heterogeneity across studies was large (I^2^ ≥93.3) in all analyses. For studies comparing knowledge gained from blended learning versus no intervention, the pooled effect size was 1.40 (95% CI 1.04-1.77; *P<*.001; n=20 interventions) with no significant publication bias, and exclusion of any single study did not change the overall result. For studies comparing blended learning with nonblended learning (pure e-learning or pure traditional face-to-face learning), the pooled effect size was 0.81 (95% CI 0.57-1.05; *P<*.001; n=56 interventions), and exclusion of any single study did not change the overall result. Although significant publication bias was found, the trim and fill method showed that the effect size changed to 0.26 (95% CI -0.01 to 0.54) after adjustment. In the subgroup analyses, pre-posttest study design, presence of exercises, and objective outcome assessment yielded larger effect sizes.

**Conclusions:**

Blended learning appears to have a consistent positive effect in comparison with no intervention, and to be more effective than or at least as effective as nonblended instruction for knowledge acquisition in health professions. Due to the large heterogeneity, the conclusion should be treated with caution.

## Introduction

Electronic learning (e-learning) has quickly become popular for health education [1-3], especially since the emergence of the Internet has allowed its potential to be realized [4]. E-learning can not only transcend space and time boundaries and improve convenience and effectiveness for individualized and collaborative learning, but also provide reusable and up-to-date information through the use of interactive multimedia [3,5-9]. However, it also suffers from disadvantages such as high costs for preparing multimedia materials, continuous costs for platform maintenance and updating, as well as learners’ feelings of isolation in virtual environments [8,10,11]. Traditional learning must be conducted at a specific time and place and is considered vital in building a sense of community [12,13]. Blended learning, defined as the combination of traditional face-to-face learning and asynchronous or synchronous e-learning [14], has been presented as a promising alternative approach for health education because it is characterized as synthesizing the advantages of both traditional learning and e-learning [8,15,16]. Moreover, blended learning has shown rapid growth and is now widely used in education [17,18].

With the introduction of blended learning, increasing research has focused on concerns about its effectiveness. Three original research articles reporting on quantitative evaluations of blended learning were published in the 1990s [19-21], and then many were published after 2000 [16,22-29]. A quantitative synthesis of these studies could inform educators and students about evidence for, and factors influencing, the effectiveness of blended learning.

Rowe et al’s systematic review reported that blended learning has the potential to improve clinical competencies among health students [30]. In another systematic review, McCutcheon et al suggested a lack of evaluation of blended learning in undergraduate nursing education [31]. Several reviews have also summarized the evaluation of e-learning in medical education, but none separated blended learning from pure e-learning [32-34]. Furthermore, these systematic reviews were limited to only some areas or branches of health education; there has been no quantitative synthesis to evaluate the effectiveness of blended learning in all professions directly related to human and animal health.

Therefore, our study aimed to identify and quantitatively synthesize all studies evaluating the effectiveness of blended learning for health professional learners who were students, postgraduate trainees, or practitioners in a profession directly related to human or animal health. We conducted two meta-analyses: the first summarized studies comparing blended learning with no intervention, and the second explored blended learning compared with nonblended learning (including pure e-learning and traditional face-to-face learning). We also aimed to explore factors that could explain differences in learning effectiveness across characteristics of participants, interventions, and study designs. Based on previous research, we hypothesized that learning outcomes would be improved through exercises, cognitive interactivity, and peer discussion [35-38]. Exercises contain cases, quizzes, self-assessment test, and other activities requiring learners to apply knowledge acquired from the course [33]. Cognitive interactivity reflects cognitive engagement required for course participation, and multiple practice exercises, essays, and group collaborative projects account for high interactivity [38]. Peer discussion includes instructor-student or peer-peer face-to-face discussion that might arise in a typical lecture, and synchronous or asynchronous online communication such as discussion boards, email, chat, or Internet conferencing [33].

## Methods

### Reporting Standards

We conducted and reported our study according to Preferred Reporting Items for Systematic Reviews and Meta-Analyses (PRISMA) guidelines [39] (see e-Table 7 in Multimedia Appendix 1) and meta-analyses of observational studies in epidemiology [40].

### Eligibility Criteria

Inclusion criteria for studies were based on the PICOS (population, intervention, comparison, outcome, and study design) framework [39]. Studies were included only if they (1) were conducted among health professional learners, (2) used a blended learning intervention in the experimental group, (3) involved a comparison of blended learning with no intervention or nonblended learning, (4) included quantitative outcomes with respect to knowledge assessed with subjective (eg, learner self-report) or objective assessments (eg, multiple-choice question knowledge test) of learners’ factual or conceptual understanding of the course, and (5) were randomized controlled trials (RCTs) or nonrandomized studies (NRSs), which are widely used in health profession education [33]. Studies in any language and of any publication type were included. Gray literature was searched in CENTRAL and ERIC.

Studies were excluded if they did not compare blended learning with nonblended learning or no intervention, did not report quantitative outcomes with respect to knowledge, used a single-group posttest-only design, were not conducted with health professional learners, evaluated pure e-learning instead of blended learning, or used the computer only for administrative purposes. Reviews, editorials, or meeting abstracts without original data were also excluded.

### Data Sources

To identify relevant studies, we conducted a search of citations in Medline, CINAHL, Science Direct, Ovid Embase, Web of Science, CENTRAL, and ERIC. Key search terms included delivery concepts (eg, blended, hybrid, integrated, computer-aided, computer-assisted; learning, training, education, instruction, teaching, course), participants’ characteristics (eg, physician*, medic*, nurs*, pharmac*, dent*, cme, health*), and study design concepts (eg, compar*, trial*, evaluat*, assess*, effect*, pretest*, pre-test, posttest*, post-test, preintervention, pre-intervention, postintervention, post-intervention). The asterisk (*) was used as a truncation symbol for searching. For instance, evaluat* retrieved entries containing the following words: evaluate, evaluation, or evaluative, etc. E-Table 1 in Multimedia Appendix 1 describes the complete search strategy for each database. The last date of search was September 25, 2014. In addition, all references of included studies were screened for any relevant articles.

### Study Selection

Using these criteria, QL and FZ independently screened all titles and abstracts and reviewed the full text of all potentially eligible abstracts. Conflicts between these reviewers were resolved through discussion with other members of the research group until a consensus was obtained.

### Data Extraction

QL and FZ developed a form (based on the Cochrane Consumers and Communication Review Group’s data extraction template), pilot-tested it on 10 randomly selected included publications, and refined it accordingly. Using the same form, data related to the following issues were extracted independently by QL and FZ: first author’s name, year of publication, country where the intervention was conducted, study design, study subjects, sample size, specific health profession of the intervention, comparison intervention, intervention duration, exercises, interactivity, peer discussion, outcome assessment, conflict of interest (whether there was a conflict of interest), and funding from company (whether funding was obtained from a source that had a direct interest in the results). Disagreements were resolved through discussion with another research team member until agreement was reached. If the required data for the meta-analyses were missing from the original report, attempts were made to obtain the information by contacting the corresponding authors by email.

### Quality Assessment

Recognizing that many nonrandomized and observational studies would be included, the methodological quality of the studies was evaluated using a modified Newcastle-Ottawa Scale (also called the Newcastle-Ottawa Scale-Education), which is an instrument used to appraise the methodological quality of original medical education research studies, typically in the process of a literature review of a field or topic in medical education [33,41-43]. Each study could receive up to 6 points and was rated in the following five domains:

- Representativeness: the intervention group was “truly” or “somewhat” representative of the average learner in this community (1 point).

- Selection: the comparison group was drawn from the same community as the experimental cohort (1 point).

- Comparability of cohorts (2 points possible): These include nonrandomized two-cohort studies (further classified into “controlled for baseline learning outcome [eg, adjusted for knowledge pretest scores; 1 point]” and “controlled for other baseline characteristics [1 point]”) and randomized studies (further classified into randomized [1 point] and allocation concealed [1 point]).
- Blinding: outcome assessment was blinded (1 point). These include (1) blinded if the assessor cannot be influenced by group assignment; (2) assessments that do not require human judgment (eg, multiple-choice tests or computer-scored performance) are considered to be blinded; (3) one-group studies are not blinded unless scoring does not require judgment or authors describe a plausible method for hiding the timing of assessment; (4) participant-reported outcomes are never blinded.
- Follow-up: subjects lost to follow-up were unlikely to introduce bias; small number lost (75% or greater follow-up) or description provided of those lost (1 point).

In addition, we evaluated the quality of evidence with the Grades of Recommendation, Assessment, Development, and Evaluation (GRADE) instrument [44-53]. GRADE identifies five factors that may decrease the quality of evidence of studies, and three factors that may increase it. RCTs start with a high rating and observational studies with a low rating. Ratings are modified downward due to (1) study limitations (risk of bias) [47], (2) inconsistency of results [50], (3) indirectness of evidence [51], (4) imprecision [49], and (5) likely publication bias [48]. Ratings are modified upward due to (1) large magnitude of effect, (2) dose response, and (3) confounders likely to minimize the effect. Evaluating these elements, we determine the quality of evidence as “high” (ie, further research is very unlikely to change our confidence in the estimate of effect), “moderate” (ie, further research is likely to have an important impact on our confidence in the estimate of effect and may change the estimate), “low” (ie, further research is very likely to have an important impact on our confidence in the estimate of effect and is likely to change the estimate), or “very low” (ie, we are very uncertain about the estimate).

### Data Synthesis

Analyses were carried out for knowledge outcomes using Stata Version 12.0 and R 3.1.2. The standardized mean difference (SMD; Hedges *g* effect sizes), converted from means and standard deviations from each study, was used [33,54]. When the mean was available but the standard deviation (SD) was not, we used the mean SD of all other included studies. As the overall scores of included studies were not the same and SMD could eliminate the effects of absolute values, we adjusted the mean and SD so that the average SD could replace the missing value of SD.

The I^2^ statistic was used to quantify heterogeneity across studies [55]. When the estimated I^2^ was equal to or greater than 50%, this indicated large heterogeneity. As the studies incorporated are functionally different and involve different study designs, participants, interventions, and settings, a random-effects model allowing more heterogeneity was used. Meta-analyses were conducted and forest plots were created. To explore publication bias, funnel plots were created and Begg’s tests were performed. To explore potential sources of heterogeneity, we performed multiple meta-regression and subgroup analyses based on factors selected in advance, such as study design, country socioeconomic status, participant type, duration of intervention, randomization, quality score, exercises, interactivity, peer discussion, outcome assessment, and intervention of the control group. Moreover, we performed sensitivity analyses to test the robustness of findings.

## Results

### Study Selection

**Figure 1:**
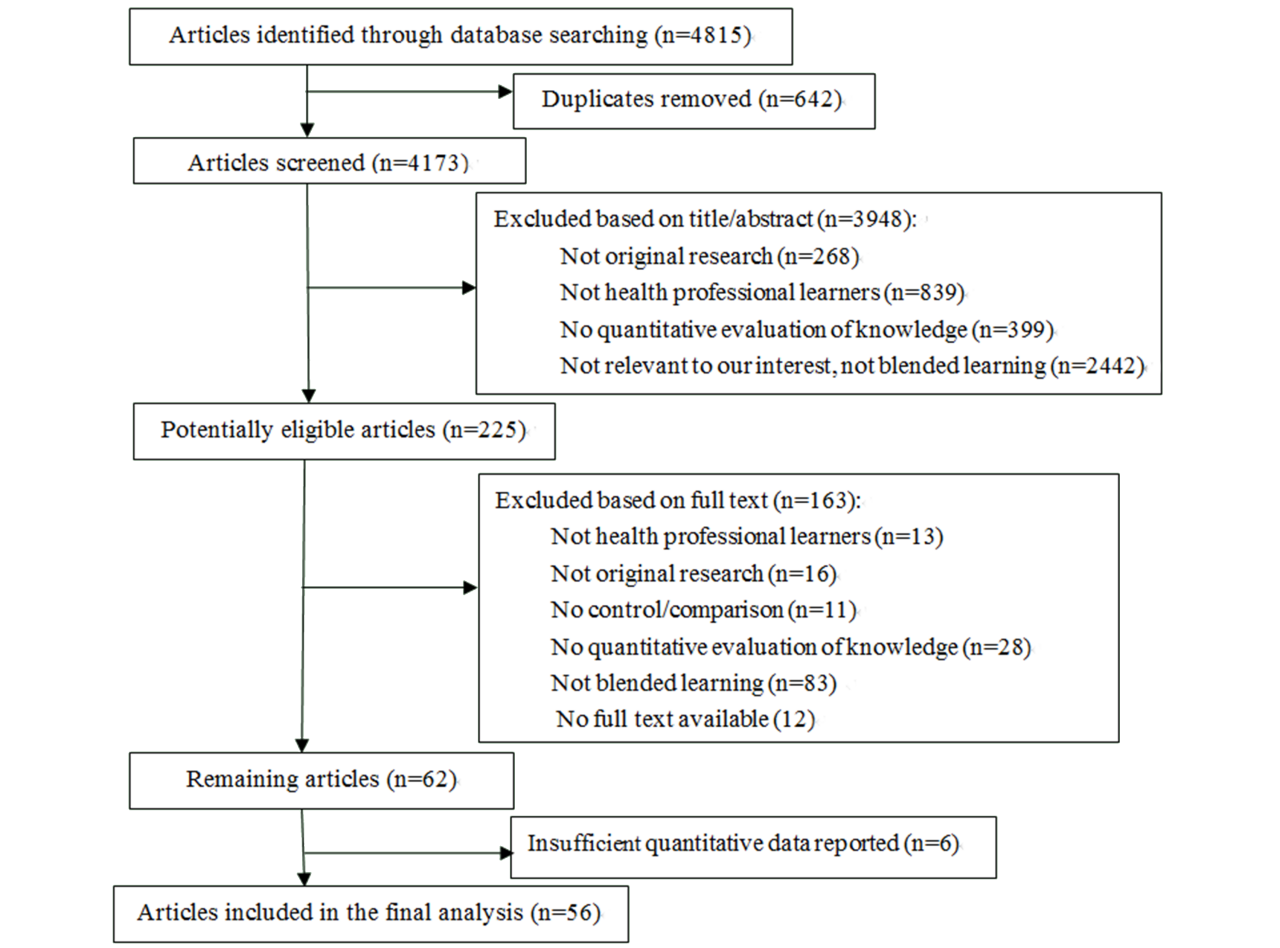
Study selction process.

The search strategy identified 4815 citations from the databases, and 642 duplicates were removed. After scanning the titles and abstracts, 225 were found to be potentially eligible. Then, full texts were read for further assessment, and 62 remained. For 12 articles without accessible full texts and 6 without sufficient quantitative data (mean knowledge scores), we tried contacting the authors by email but received no reply. Thus, 56 publications were included, among which one publication compared blended learning with both no intervention and nonblended instruction (Figure 1). No more relevant articles were found by reviewing the references of the included articles. E-Table 2 in Multimedia Appendix 1 includes the references of articles excluded based on full text (n=163) and insufficient quantitative data reported (n=6).

### Study Characteristics

In the meta-analysis, we included 13 publications representing 20 interventions published from 2004-2014, which compared blended learning with no intervention and included 2238 health professional participants [22-24,56-65]. The number of participants ranged from 6 [61] to 817 [62], and the duration of the intervention ranged from 24 hours [63] to one semester [58].

We included 44 publications representing 56 interventions comparing blended learning with nonblended learning published from 1991 to 2014 that covered 6110 health profession participants [16,19-21,25,26,28,29,63,66-100]. There was 1 pre-posttest one-group intervention, 27 posttest-only two-group interventions, and 28 pre-posttest two-group interventions. The number of participants ranged from 14 [72] to 609 [84], and the duration ranged from 1 hour [101] to 1 year [77].

Components or features of the study intervention were mostly “Web-based+ face-to-face”, “e-learning+ class session”, and “Web-based online instruction+ off-line instruction (review of the core contents on the online program, case analysis, small group discussion, and miscellaneous activities)”. “Modality or technology” varied, such as “Moodle, on-site workshops”, “asynchronous discussion forums, a live audio and text-based online synchronous session (Centra); online modules (Macromedia Breeze)”. More than 80% of the interventions were measured using objective assessment, which included multiple choice questions, true or false questions, matching questions, and essays. For most studies, there was no delay between the end of the intervention and the posttest. Table 1 summarizes the key features and e-Table 3 in Multimedia Appendix 1 describes the detailed information.

**Table 1:** Summary description of included studies.

Study characteristics		No intervention comparison		Nonblended learning comparison	
		Interventions, n (%) (N=20)	Participants, n (N=2238)	Interventions, n (%) (N=56)	Participants, n (N=6110)
**Study design**					
	Pre-posttest 1-group	17 (85.0)	1656	27 (48.2)	97
	Posttest 2-group	2 (10.0)	130	28 (50.0)	3468
	Pre-posttest 2-group	1 (5.0)	452	1 (1.8)	2545
**RCT/NRS**					
	RCT	2 (10.0)	130	31 (55.4)	2919
	NRS	18 (90.0)	2108	25 (44.6)	3191
**Country**					
	Developed	14 (70.0)	1673	44 (78.6)	4489
	Developing	6 (30.0)	565	12 (21.4)	1621
**Participant**					
	Medical students	9 (45.0)	887	37 (66.1)	4593
	Nursing students	1 (5.0)	69	9 (16.1)	870
	Nurses	2 (10.0)	103	5 (8.9)	259
	Physicians	6 (30.0)	137	2 (3.6)	256
	Public health workers	1 (5.0)	817	1 (1.8)	66
	Others	1 (5.0)	225	1 (1.8)	66
**Intervention duration**					
	˂1 semester	17 (85.0)	2038	43 (76.8)	4578
	≥1 semester	3 (15.0)	200	13 (23.2)	1532
**Exercises**					
	Present	15 (75.0)	1273	41 (73.2)	4526
	Absent	5 (25.0)	965	15 (26.8)	1584
**Interactivity**					
	High	15 (75.0)	1559	35 (62.5)	4460
	Low	5 (25.0)	679	21 (37.5)	1650
**Peer discussion**					
	Present	10 (50.0)	1456	28 (50.0)	3369
	Absent	10 (50.0)	782	28 (50.0)	2741
**Outcome assessment**					
	Objective	16 (80.0)	1833	53 (93.6)	5832
	Subjective	4 (20.0)	405	3 (6.4)	278
**Comparison intervention**					
	E-learning	NA	NA	5 (8.9)	205
	Traditional learning	NA	NA	51 (91.1)	5905
**Conflict of interest**					
	Yes	0	0	2 (3.6)	612
	No	20 (100.0)	2238	54 (96.4)	5498
**Quality score**					
	≥4	5 (25.0)	730	47 (83.9)	4965
	˂4	15 (75.0)	1508	9 (16.1)	1145

### Study Quality

All of the intervention groups in the included studies were representative of average learners. Ten percent (2/20) of no-intervention controlled studies and 98% (55/56) of nonblended learning controlled studies selected the control group from the same community as the experimental group. Nearly a third (30%, 6/20) of the no-intervention controlled studies and 46% (26/56) of nonblended learning controlled studies reported blinded outcome assessment. All of the no-intervention controlled studies (100%) and 96% (54/56) of nonblended learning controlled studies reported completeness of follow-up. The mean (SD) quality score was 3.40 (0.82) for no-intervention controlled studies, and 4.45 (0.78) for nonblended learning controlled studies. The results of the quality assessment are shown in e-Table 4 in Multimedia Appendix 1.

### Quantitative Data Synthesis

#### Comparisons With No Intervention

**Figure 2:**
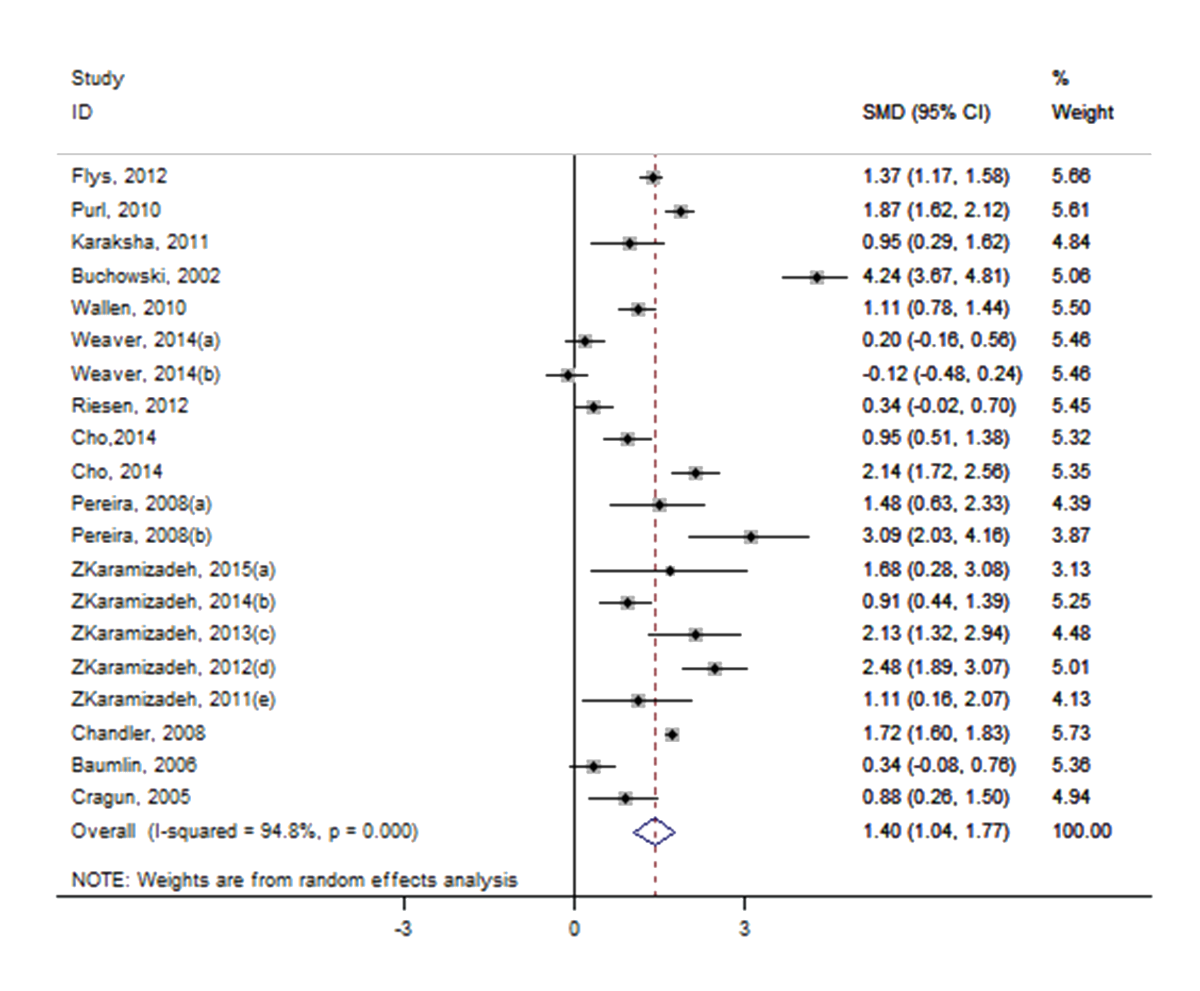
Forest plot of blended learning versus no intervention.

**Figure 3:**
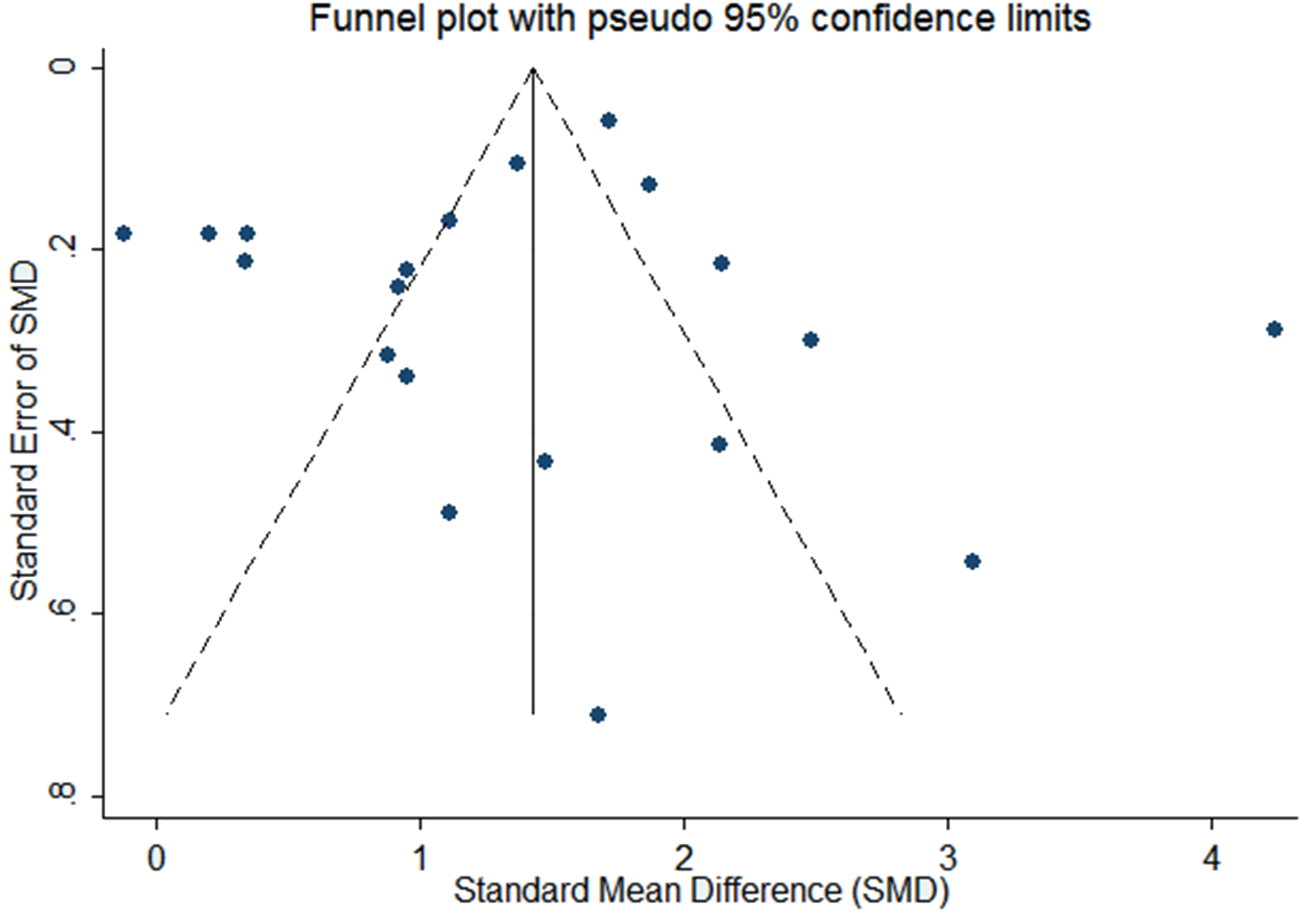
Funnel plot of blended learning versus no intervention.

As effect sizes larger than 0.8 were considered to be large [102], the pooled effect size (SMD 1.40; 95% CI 1.04-1.77; *Z*=7.52, *P*<.001) suggests a significantly large effect. However, significant heterogeneity was observed among studies (*P*<.001, I^2^=94.8%, 95% CI 93.1-96.0), and individual effect sizes ranged from -0.12 to 4.24. Figure 2 shows detailed results of the meta-analysis. The test of funnel plots (Figure 3) indicated no significant publication bias among studies (Begg’s test *P*=.587). Based on risk of bias and large effect, we graded the quality of evidence as moderate. E-Table 5 in Multimedia Appendix 1 provides the GRADE evidence profile. E-Table 6 in Multimedia Appendix 1 contains the mean, standard difference, and number of participants for both blended learning and no intervention/nonblended learning.

#### Meta-Regression and Subgroup Analysis

We investigated a multiple regression model with each possible source of heterogeneity (I^2^_res=85.33%, adjusted R^2^=48.89%; I^2^_res means residual variation due to heterogeneity) and found that the outcome assessment (*P*=.03) was a potential source of heterogeneity (Table 2). Studies with objective outcome assessments had larger pooled effect sizes. Furthermore, subgroup analyses were performed to evaluate the sources of heterogeneity. A statistically significant interaction favoring pre-posttest two-groups designs and pre-posttest one-group designs was found (*P* for interaction<.001), which was consistent with the result of the meta-regression. Statistical differences existed between the groups of participants (*P* for interaction<.001). Nonrandomized studies had larger effects than randomized ones (*P* for interaction=.01). The effect size was significantly larger for blended learning with objective assessment than with subjective assessment (*P* for interaction=.005). However, we did not find support for the hypotheses regarding subgroup interactions across levels of exercises (*P* for interaction=.92).

#### Sensitivity Analyses

Exclusion of any single study did not change the overall result, which ranged from 1.24 (95% CI 0.91-1.57) to 1.48 (95% CI 1.14-1.83).

#### Comparisons With Nonblended Learning

**Figure 4:**
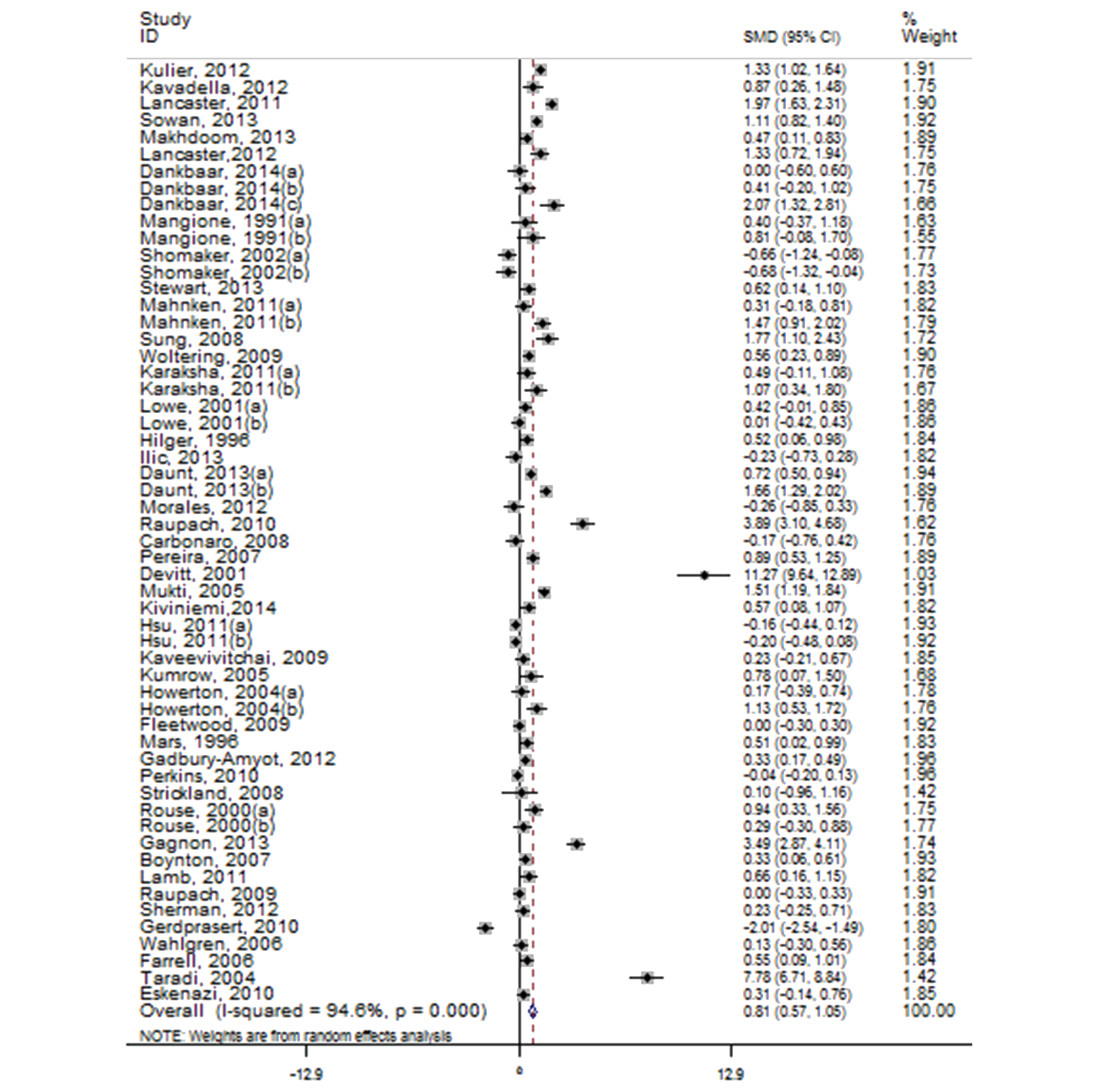
Forest plot of blended learning versus non-blended learning.

**Figure 5:**
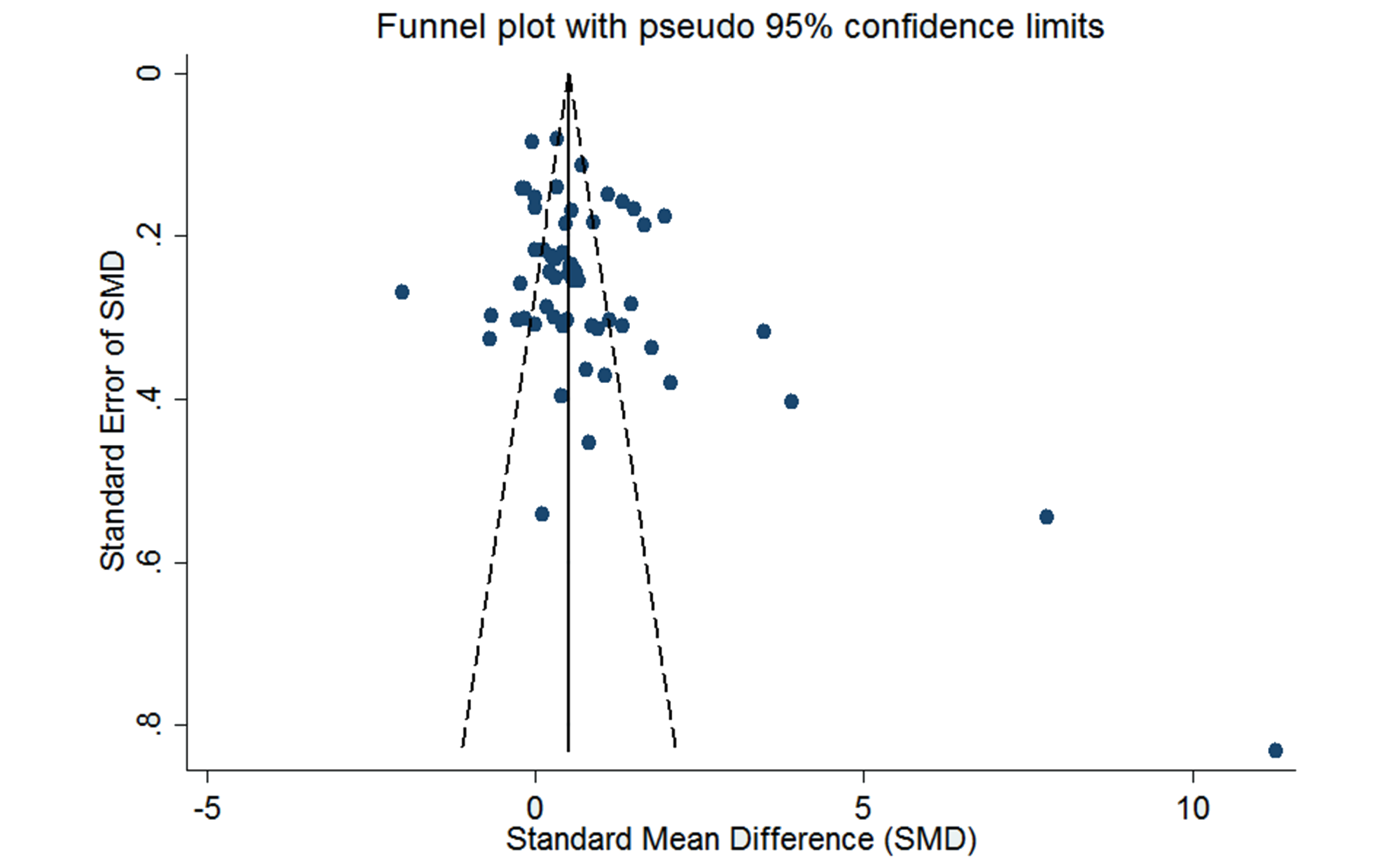
Funnel plot of blended learning versus non-blended learning.

The pooled effect size (SMD 0.81; 95% CI 0.57-1.05; *Z*=6.59, *P*<.001) significantly reflected a large effect, and significant heterogeneity was observed among studies (*P<*.001, I^2^=94.6%, 95% CI 93.7-95.5). Figure 4 shows detailed results of the main analysis. The test of asymmetry funnel plot (Figure 5) indicated publication bias among studies (Begg’s test *P*=.01). The publication bias may have been towards larger studies with generally large magnitudes of effects. The trim and fill method indicated that the effect size changed to 0.26 (95% CI -0.01 to 0.54) after adjusting for publication bias, which suggested that blended learning was at least as effective as nonblended learning. Based on risk of bias, publication bias, and large effect, we graded the quality of evidence as low. E-Table 5 in Multimedia Appendix 1 provides the GRADE evidence profile.

**Table 2:** Subgroup analysis of blended learning versus no intervention.

Subgroup		Interventions, n	Pooled effect sizes (95% CI)	Heterogeneity (I^2^), *P*	Interaction, *P* ^a^	Meta-regression	
						Coef.	*P*
All interaction		20	1.40 (1.04-1.77)	94.8% (93.1-96.0), *P*<.001			
**Study design**							
	Posttest 2-groups	2	0.59 (0.00-1.18)	57.0%, *P*=.13			
	Pre-posttest 1 group	17	1.47 (1.05-1.88)	95.0% (93.3-96.3), *P*<.001	<.001	.27	.81
	Pre-posttest 2-groups	1	1.87 (1.62-2.13)	0			
**Country**							
	Developed	14	1.29 (0.83-1.75)	96.0% (94.6-97.1), *P*<.001	.23	-.22	.90
	Developing	6	1.71 (1.20-2.22)	76.5% (47.4-89.5), *P*=.001			
**Participant**							
	Medical students	9	1.13 (0.32-1.94)	96.8% (95.4-97.8), *P*<.001			
	Nursing students	1	2.14 (1.72-2.56)	0			
	Nurses	2	1.05 (0.79-1.91)	0.0%, *P*=.56	<.001	.05	.82
	Physicians	6	1.84 (1.14-2.54)	81.2% (59.7-91.2), *P*<.001			
	Public health workers	1	1.72 (1.60-1.83)	0			
	Others	1	1.37 (1.17-1.58)	0			
**Intervention duration**							
	˂1 semester	17	1.39 (1.10-1.18)	89.2% (84.2-92.6), *P*<.001	.97	-.33	.69
	≥1 semester	3	1.43 (-0.82-3.68)	98.9% (98.1-99.3), *P*<.001			
**Randomization**							
	Randomized	2	0.59 (.001-1.64)	57.0%, *P*=.013	.01	.67	.45
	Nonrandomized	18	1.49 (1.11-1.87)	94.9% (93.2-96.2), *P*<.001			
**Quality score**							
	≥4	5	1.89 (1.13-2.66)	96.2% (93.4-97.8), *P*<.001	.63	-1.05	.29
	˂4	15	1.23 (.77-1.69)	94.3% (92.1-95.9), *P*<.001			
**Exercises**							
	Present	10	1.28 (0.64-1.90)	95.1% (93.2-96.4), *P*<.001	.92	-.21	.75
	Absent	10	1.53 (1.08-1.99)	89.5% (88.7-96.7), *P*<.001			
**Interactivity**							
	High	15	1.54 (1.07-2.00)	95.6% (94.0-96.7), *P*<.001	.20	-1.25	.41
	low	5	1.05 (0.44-1.65)	90.9% (81.7-95.5), *P*<.001			
**Peer discussion**							
	Present	10	1.25 (0.70-1.79)	96.2% (94.2-97.2), *P*<.001	.11	-.07	.97
	Absent	10	1.87 (1.21-2.53)	93.1% (88.6-95.3), *P*<.001			
**Outcome assessment**							
	Objective	16	1.66 (1.29-2.04)	91.9% (88.4-94.3), *P*<.001	.005	-2.02	.03
	Subjective	4	0.46 (-0.30-1.22)	95.8% (92.1-97.8), *P*<.001			
**Funding from company**							
	Yes	2	2.29 (-1.53 to 6.11)	99.2%, *P*<.001	.61	-.93	.37
	No	18	1.30 (.97-1.62)	92.7% (88.9-94.7), *P*<.001			

^a^ *P* for interaction means the *P* of heterogeneity between groups.

#### Meta-Regression and Subgroup Analysis

A multiple regression model for each possible source of heterogeneity was conducted (I^2^_res=94.59%, adjusted R^2^=-26.38%), and no significant source of heterogeneity was found (Table 3). Furthermore, subgroup analyses were performed to evaluate the sources of heterogeneity. We found both pre-posttest two-group studies and pre-posttest one-group studies showed larger effects than posttest-only studies (*P* for interaction<.001). It was shown that the presence of exercises could yield a larger SMD (*P* for interaction=.49). Studies with objective assessments yielded a larger effect than studies with subjective assessments (*P* for interaction=.01). Studies without conflicts of interest yielded a larger effect than those with conflicts of interest (*P* for interaction<.001). However, high interactivity and presence of peer discussion did not yield larger effect sizes (*P* for interaction>.85).

#### Sensitivity Analyses

Exclusion of any single study did not change the overall result, which ranged from 0.70 (95% CI 0.48-0.92) to 0.86 (95% CI 0.63-1.10).

## Discussion

### Principal Findings

This meta-analysis shows that blended learning has a large consistent positive effect (SMD 1.40, 95% CI 1.04-1.77) on knowledge acquisition compared with no intervention, which suggested that blended learning was very effective and educationally beneficial in health professions. Moreover, we also found that blended learning had a large effect (SMD 0.81, 95% CI 0.57-1.05) in comparison with the nonblended learning group. This means that blended learning may be more effective than nonblended learning, including both traditional face-to-face learning and pure e-learning. Possible explanations could be as follows: (1) compared with traditional learning, blended learning allows students to review electronic materials as often as necessary and at their own pace, which likely enhances learning performance [8,16], and (2) compared with e-learning, blended learning learners are less likely to experience feelings of isolation or reduced interest in the subject matter [8,11,103]. However, publication bias was found in the nonblended learning comparison group, and the trim and fill method showed that the pooled effect size changed to 0.26 (-0.01 to 0.54), which means blended learning is at least as effective as nonblended learning. To the best of our knowledge, this may be the first meta-analysis to reveal the effectiveness of blended learning for knowledge acquisition in health professions, which includes all those directly related to human and animal health.

However, large heterogeneity was found across studies in both no-intervention and nonblended comparisons, and the subgroup comparisons partially explained these differences. The heterogeneity may be due to variations in study design, outcome assessment, exercises, conflict of interest, randomization, and type of participants. We found that effect sizes were significantly higher for studies using pre-posttest designs than posttest-only designs, which suggested that the former improved learning outcomes relative to the latter. As pretests may inform instructors about the knowledge learners have acquired before the course, which is considered to be one of the most important factors influencing education [104], they allow instructors to determine learning objectives and to prepare course materials accordingly [105]. Therefore, it is necessary for educators to administer pretests to learners to prepare well for courses. We also found that studies with objective assessments yielded a larger effect than those with subjective assessments. In contrast, Cook et al reported no difference between objective and subjective assessments in knowledge scores [33]. This is probably due to differences in personality traits of learners, as people with greater confidence tend to give higher ratings on subjective assessments than people who are less confident [106]. Thus, educators should objectively assess learners instead of using subjective evaluations.

**Table 3:** Subgroup analysis of blended learning versus nonblended learning.

Subgroup		Interventions, n	Pooled effect sizes (95% CI)	Heterogeneity (I^2^), *P*	Interaction, *P*	Meta-regression	
						Coef.	*P*
All interventions		56	0.81 (0.57-1.05)	94.6% (93.7-95.5), *P*<.001			
**Study design**							
	Posttest 2-groups	27	0.70 (0.32-1.07)	94.0% (92.3-95.3), *P*<.001	<.001		
	Pre-posttest 2-groups	28	.89 (0.58-1.19)	94.5% (93.0-95.6), *P*<.001		-.001	.99
	Pre-posttest 1-group	1	1.97 (1.63-2.32)	0			
**Country**							
	Developed	44	0.80 (0.54-1.01)	93.2% (91.7-94.4), *P*<.001	.83	.13	.86
	Developing	12	0.87 (0.22-1.53)	97.2% (96.2-97.9), *P*<.001			
**Participant**							
	Medical students	38	0.88 (0.60-1.17)	94.8% (93.6-95.7), *P*<.001			
	Nursing students	9	0.42 (-0.32-1.16)	96.0% (94.0-97.3), *P*<.001			
	Nurses	5	0.87 (0.09-1.65)	87.7% (73.8-94.2), *P*<.001	.03	-.17	.61
	Physicians	2	1.33 (1.05-1.60)	0.0%, *P*=.996			
	Public health workers	1	0.57 (0.08-1.07)	0			
	Others	1	0.66 (0.16-1.15)	0			
**Intervention duration**							
	˂1 semester	43	0.73 (0.45-1.00)	94.5% (93.3-95.5), *P*<.001	.17	-.29	.68
	≥1 semester	13	1.10 (0.63-1.59)	93.9% (91.3-95.8), *P*<.001			
**Randomization**							
	Randomized	31	0.75 (0.38-1.12)	95.1% (94.0-96.1), *P*<.001	.63	.29	.69
	Nonrandomized	25	0.87 (0.56-1.05)	94.1% (92.3-95.4), *P*<.001			
**Quality score**							
	≥4	47	0.82 (0.55-1.09)	94.9% (93.9-95.8), *P*<.001	.99	-.27	.78
	˂4	9	0.83 (0.39-1.26)	90.4% (84.1-94.2), *P*<.001			
**Exercises**							
	Present	41	0.93 (0.63-1.25)	95.7% (94.9-96.4), *P*<.001	.49	-.51	.51
	Absent	15	0.53 (0.26-0.80)	82.5% (72.2-88.9), *P*=0.011			
**Interactivity**							
	High	37	0.84 (0.55-1.13)	95.2% (94.2-96.1), *P*<.001	.85	.48	.60
	Low	19	0.78 (0.35-1.23)	93.4% (91.2-95.1), *P*<.001			
**Peer discussion**							
	Present	28	0.82 (0.46-1.18)	95.9% (94.9-96.7), *P*<.001	.93	-.43	.96
	Absent	28	0.80 (0.48-1.12)	92.7% (90.6-94.4), *P*<.001			
**Outcome assessment**							
	Objective	53	0.85 (0.61-1.10)	94.8% (93.8-95.6), *P*<.001	.01	-.91	.47
	Subjective	3	0.07 (-0.46 to 0.60)	68.6% (0-90.9), *P*=.04			
**Comparison intervention**							
	E-learning	5	0.40 (-0.21-1.01)	77.5% (34.8-87.8), *P*=.23	.17	.69	.52
	Traditional learning	51	0.85 (0.60-1.11)	95.0% (94.1-95.8), *P*<.001			
**Conflict of interest**							
	Yes	2	-0.06 (-0.21 to 0.10)	0.0%	<.001	1.17	.44
	No	54	0.85 (0.60-1.10)	94.5% (93.5-95.4), *P*<.001			

Additionally, effect size was found to be significantly larger for blended courses with exercises versus no exercises, which was consistent with the results of a previous study conducted by Cook et al in 2006, which found that continuity clinics had higher test scores when using a question format compared to a standard format [37]. Thus, it is necessary for educators to include exercises in their teaching, such as cases and self-assessment questions. However, we failed to confirm our hypothesis that presence of peer discussion and high interactivity would yield larger effect sizes. Although we found statistical differences between the RCTs and NRS in the no-intervention comparison, it could probably be due to chance as there were only two RCTs (130 participants) included. Differences between studies with conflicts of interest and those without conflicts of interest in nonblended comparisons could be also due to chance, as only two studies with conflicts of interest (612 participants) were included. The remainder of the high heterogeneity may arise from other characteristics, such as individual learning styles, study intervention, assessment instrument, and ongoing access to learning materials [33,107,108], for which detailed information was not available in the included studies. As Wong et al cited in their review, different modes of course delivery suit different learners in different environments [109].

Our samples consisted of various health professional learners (nurses, medical students, nursing students, physicians, public health workers, and other health professionals) across a wide variety of health care disciplines, such as medicine, nursing, ethics, health policy, pharmacy, radiology, genetics, histology, and emergency preparedness. Moreover, we found medium or large effects for the pooled effect sizes of almost all subgroup analyses exploring variations in study design, participant type, randomization, quality scores, exercises, interactivity, and peer discussion. Thus, our results suggest that health care educators should use blended learning as a teaching component in various disciplines and course settings.

### Strengths and Limitations

Our meta-analysis also has several strengths. Evaluations of the effectiveness of blended learning for health professions are timely and very important for both medical educators and learners. We intentionally kept our scope broad in terms of subjects and included all studies with learners from health professions. We searched for relevant studies in manifold research databases up to September 2014. The systematic literature search encompassed multiple databases and had few exclusion criteria. We also conducted all aspects of the review process in duplicate.

However, there are limitations to consider. First, although we searched gray literature in two databases (CENTRAL and ERIC), gray literature indexed by other databases may have been missed, which could be the reason for the observed publication bias. Second, the quality of meta-analyses is dependent on the quality of data from the included studies. Although the standard deviation of eight interventions was not available due to poor reporting, we used the average standard deviation of other included studies and imputed effect sizes with concomitant potential for error. Third, despite conducting the review and extraction independently and in duplicate, the process was subjective and dependent on the descriptions of the included articles instead of direct evaluation of interventions. Fourth, although the modified Newcastle–Ottawa scale is a useful and reliable tool for appraising methodological quality of medical education research and enhances flexibility for different study designs, it increases the risk of reviewer error or bias due to a certain amount of rater subjectivity. Then, results of subgroup analyses should be interpreted with caution because of the absence of a priori hypotheses in some cases, such as study design, country socioeconomic status, and outcome assessment. Moreover, although the subgroup analyses showed the variability of participant types, socioeconomic status of country, intervention duration, interactivity, peer discussion, and study design of RCT or NRS did not make a difference in the overall results, the large clinical heterogeneity and inconsistent magnitude of effects across studies makes it difficult to generalize the conclusions. In addition, as variability of study interventions, assessment instruments, circumstances and so on, which were not assessed, could be potential sources of heterogeneity, the results of both meta-analyses should be treated with caution. Furthermore, publication bias was found in the meta-analysis with the nonblended comparison. Although we used the trim and fill method for adjustment, the results should be treated with caution.

### Implications

Our study has implications for both research on blended learning and education in health professions. Despite the fact that conclusions could be weakened by heterogeneity across studies, the results of our quantitative synthesis demonstrated that blended learning may have a positive effect on knowledge acquisition across a wide range of learners and disciplines directly related to health professions. In summary, blended learning could be promising and worthwhile for further application in health professions. The difference in effects across subgroup analyses indicates that different methods of conducting blended courses may demonstrate differing effectiveness. Therefore, researchers and educators should pay attention to how to implement a blended course effectively. This question could be answered successfully through studies directly comparing different blended instructional methods. Thus, such studies are of critical importance.

Studies comparing blended learning with no intervention suggested that blended learning in health professions might be invariably effective. However, although observational studies yielded a large effect size, the quality of evidence was lower due to their inherent study design limitations. Additionally, owing to the small number of RCTs, the meta-analysis did not meet the optimal size (imprecision) and therefore, quality of evidence was ranked lower. Thus, despite the consistency of effect and no significant reporting bias, the evidence of the no-intervention comparison was of moderate quality, which means further research is likely to have an impact on our confidence in the estimate of effect and may change the estimate, and RCTs with large samples may modify the estimates. Thus, there is still great value in further research comparing blended learning with no intervention, and RCTs with large samples may modify the estimates. For nonblended comparisons, pooled estimates showed that blended learning is more effective than or at least as effective as pure e-learning and pure traditional learning. However, due to publication bias towards larger studies with generally large magnitudes of effects, the evidence was of low quality, which means further research is very likely to change our estimate. Furthermore, only four studies using e-learning were included. Therefore, the effect of blended learning especially in comparison with e-learning should be evaluated in future research, and studies with small magnitudes of effect should merit publication.

### Conclusions

Blended learning appears to have a consistent positive effect in comparison with no intervention and appears to be more effective than or at least as effective as nonblended instruction for knowledge acquisition in health professions. Moreover, pre-posttest study design, presence of exercises, and objective outcome assessment in blended courses could improve health care learners’ knowledge acquisition. Due to the large heterogeneity, the conclusion should be treated with caution.

## Appendix

Multimedia Appendix 1 E-tables 1-7.

## Abbreviations

GRADE: Grades of Recommendation, Assessment, Development, and Evaluation
PICOS: population, intervention, comparison, outcome, and study design
PRISMA: Preferred Reporting Items for Systematic Reviews and Meta-Analyses
SD: standard deviation
SMD: standardized mean difference
