# Supplementary material for: The Effectiveness of Blended Learning in Health Professions: Systematic Review and Meta-Analysis
Source: J Med Internet Res. 2016 Jan 4;18(1):e2. doi: 10.2196/jmir.4807 (PMC4717286; doi:10.2196/jmir.4807)
Supplement: Multimedia Appendix 1 [file jmir_v18i1e2_app1.pdf]

Multimedia Appendix 1. E-tables.

E-table 1.search strategy

Search date 2014.9.25

***=wild word   Adj=adjacent**

Medline:

1.“blended learning”[Title/Abstract] OR “hybrid learning”[Title/Abstract] OR “integrated learning”[Title/Abstract] OR “computer-aided learning”[Title/Abstract] OR “computer-assisted learning”[Title/Abstract] OR “distributed learning”[Title/Abstract] OR “hybrid training”[Title/Abstract] OR “integrated training”[Title/Abstract] OR “computer-aided training”[Title/Abstract] OR “integrated education”[Title/Abstract] OR “computer-aided education”[Title/Abstract] OR "computer-assisted education"[Title/Abstract] OR "distributed education"[Title/Abstract] OR “integrated instruction”[Title/Abstract] OR “computer-aided instruction”[Title/Abstract] OR "computer-assisted instruction"[Title/Abstract] OR “blended teaching”[Title/Abstract] OR “integrated teaching”[Title/Abstract] OR “computer-aided teaching”[Title/Abstract] OR “computer-assisted teaching”[Title/Abstract] OR “blended course”[Title/Abstract] OR “hybrid course”[Title/Abstract] OR “integrated course”[Title/Abstract] OR“computer-assisted course”[Title/Abstract]
2.physician*[Title/Abstract] OR medic*[Title/Abstract] OR nurs*[Title/Abstract] OR pharmac*[Title/Abstract] OR dental[Title/Abstract] OR health*[Title/Abstract] OR cme[Title/Abstract]
3.compar* OR trial* OR evaluat* OR assess* OR effect* OR pretest* OR pre-test OR posttest* OR post-test OR preinterven* OR pre-intervention OR postinterven* OR post-intervention
4.1 AND 2 AND 3

Ovid Embase:

1.(blended OR hybrid OR integrated OR distributed OR computer-aided OR computer-assited) adj (learing OR training OR educat* OR instruct* OR teach* OR course*).ti,ab.
2.(physician*OR medic* OR nurs* OR pharmac* OR dental OR health* OR cme) .ab.
3.(compar* OR trial* OR evaluat* OR assess* OR effect* OR pretest* OR pre-test OR posttest* OR post-test OR preintervention OR pre-intervention OR

postintervention OR post-intervention).af.
4. 4.1 AND 2 AND 3

Web of science

1.title: (“blended learning” OR “hybrid learning” OR “integrated learning” OR “computer-aided learning” OR “computer-assisted learning” OR “distributed learning” OR “hybrid training” OR “integrated training” OR “computer-aided training” OR “integrated education” OR “computer-aided education” OR "computer-assisted education" OR "distributed education" OR “integrated instruction” OR “computer-aided instruction” OR "computer-assisted instruction" OR “blended teaching” OR “integrated teaching” OR “computer-aided teaching” OR “computer-assisted teaching” OR“blended course” OR“hybrid course” OR “integrated course” OR“computer-assisted course”)
2.subject: (physician*OR medic* OR nurs* OR pharmac* OR dental OR cme OR health*)
3. subject: (compar* OR trial* OR evaluat* OR assess* OR effect* OR pretest* OR pre-test OR posttest* OR post-test OR preintervention OR pre-intervention OR postintervention OR post-intervention)
4. 1 AND 2 AND 3

CINAHL

1.TI“blended learning” OR TI“hybrid learning” OR TI“integrated learning” OR TI“computer-aided learning” OR TI“computer-assisted learning” OR TI“distributed learning” OR TI“hybrid training” OR TI“integrated training” OR TI“computer-aided training” OR TI“integrated education” OR TI“computer-aided education” OR TI"computer-assisted education" OR TI"distributed education" OR TI“integrated instruction” OR TI“computer-aided instruction” OR TI"computer-assisted instruction" OR TI“blended teaching” OR TI“integrated teaching” OR TI“computer-aided teaching” OR TI“computer-assisted teaching” OR TI“blended course” OR TI“hybrid course” OR TI“integrated course” OR TI“computer-assisted course” OR AB“blended learning” OR AB“hybrid learning” OR AB“integrated learning” OR AB“computer-aided learning” OR AB“computer-assisted learning” OR AB“distributed learning” OR AB“hybrid training” OR AB“integrated training” OR AB“computer-aided training” OR AB“integrated education” OR AB“computer-aided education” OR AB"computer-assisted education" OR AB"distributed education" OR AB“integrated instruction” OR AB“computer-aided instruction” OR AB"computer-assisted instruction" OR AB“blended teaching” OR AB“integrated teaching” OR AB“computer-aided teaching” OR AB“computer-assisted teaching” OR AB“blended course” OR AB“hybrid course” OR AB“integrated course” OR AB“computer-assisted course”
2. TI physician*OR TI medic* OR TI nurs* OR TI pharmac* OR TI dental OR TI health* OR TI cme OR AB physician* OR AB medic* OR AB nurs* OR AB pharmac* OR AB dental OR AB health* OR AB cme
3. compar* OR trial* OR evaluat* OR assess* OR effect* OR pretest* OR pre-test OR posttest* OR post-test OR preinterven* OR pre-intervention OR postinterven* OR post-intervention
4.1 AND 2 AND 3

ERIC

1 title:(“blended learning” OR “hybrid learning” OR “integrated learning” OR “computer-aided learning” OR “computer-assisted learning” OR “distributed learning” OR “hybrid training” OR “integrated training” OR “computer-aided training” OR “integrated education” OR “computer-aided education” OR "computer-assisted education" OR "distributed education" OR “integrated instruction” OR “computer-aided instruction” OR “computer-assisted instruction” OR “blended teaching” OR “integrated teaching” OR “computer-aided teaching” OR “computer-assisted teaching” OR “blended course” OR “hybrid course” OR “integrated course” OR “computer-assisted course”) AND abstract:(physician*OR medic* OR nurs* OR pharmac* OR dental OR health* OR cme) AND (compar* OR trial* OR evaluat* OR assess* OR effect* OR pretest* OR pre-test OR posttest* OR post-test OR preintervention OR pre-intervention OR postintervention OR post-intervention)

Sciencedirect

1.(ttl(“blended learning”) OR ttl(“hybrid learning”) OR ttl(“integrated learning”) OR ttl(“computer-aided learning”) OR ttl(“computer-assisted learning”) OR ttl(“distributed learning”) OR ttl(“hybrid training”) OR ttl(“integrated training”) OR ttl(“computer-aided training”) OR ttl(“integrated education”) OR ttl(“computer-aided education”) OR ttl("computer-assisted education") OR ttl("distributed education") OR ttl(“integrated instruction”) OR ttl(“computer-aided instruction”) OR ttl(“computer-assisted instruction”) OR ttl(“blended teaching”) OR ttl(“integrated teaching”) OR ttl(“computer-aided teaching”) OR ttl(“computer-assisted teaching”) OR ttl(“blended course”) OR ttl(“hybrid course”) OR ttl(“integrated course”) OR ttl(“computer-assisted course”))AND (tak(physician*) OR tak(medic*) OR tak(nurs*) OR tak(pharmac*) OR tak(dental) OR tak(health*) OR tak(cme))AND (compar* OR trial* OR evaluat* OR assess* OR effect* OR pretest* OR posttest* OR preintervention OR postintervention)

Cochrane Central

1. Title, Abstract, Keywords:("blended learning" OR "hybrid learning" OR "integrated learning" OR "computer-aided learning" OR "computer-assisted learning" OR "distributed learning" OR "hybrid training" OR "integrated training" OR "computer-aided training" OR "integrated education" OR "computer-aided education" OR "computer-assisted education" OR "distributed education" OR "integrated instruction" OR "computer-aided instruction" OR "computer-assisted instruction" OR "blended teaching" OR "integrated teaching" OR "computer-aided teaching" OR "computer-assisted teaching" OR "blended course" OR "hybrid course" OR "integrated course" OR "computer-assisted course")
2. Title, Abstract, Keywords: (physician*OR medic* OR nurs* OR pharmac* OR dental OR health* OR cme)
3. Search all text (compar* OR trial* OR evaluat* OR assess* OR effect* OR pretest* OR pre-test OR posttest* OR post-test OR preintervention OR pre-intervention OR postintervention OR post-intervention)
4. 1 AND 2 AND 3

E-Table 2. Reference

Section 1. Articles excluded based on full texts (n=163)

1. Aho NB, The effect of instructional design, mathematics anxiety, and attitude toward computer-assisted instruction on mathematics scores of nursing students.. 1992, UNIVERSITY OF AKRON. p. 160.
2. Andrews PV, Schwarz JHelme RD. Students can learn medicine with computers. Evaluation of an interactive computer learning package in geriatric medicine. Med J Aust 1992. 157(10): 693-695.
3. Antonoff MB, Whitson BA, Green CA, Maddaus MAD'Cunha J. Preparation of the senior medical student for integrated surgery training: Significant impact of a competency-based course. J Am Coll Surgeons 2011. 213(3, Supplement): S124.
4. ÃztÃ Rk DBulut HL. Using Computer Assisted Learning in Nursing Education: A Pilot Study in Turkey. International Journal of Caring Sciences 2012. 5(3): 302-310.
5. Bains M, Reynolds PA, McDonald FSherriff M. Effectiveness and acceptability of face-to-face, blended and e-learning: a randomised trial of orthodontic undergraduates. Eur J Dent Educ 2011. 15(2): 110-117.
6. Baumlin KM, Bessette MJ, Lewis CRichardson LD. EMCyberSchool: an evaluation of computer-assisted instruction on the Internet. Acad Emerg Med 2000. 7(8): 959-962.
7. Baumlin KM, Bessette M, Lewis CRichardson L. The emergency medicine CyberSchool: Computer-assisted instruction on the internet as a useful tool for educating medical students. Ann Emerg Med 1999. 34(4, Part 2): S70.
8. Beale EG, Tarwater PMLee VH. A retrospective look at replacing face-to-face embryology instruction with online lectures in a human anatomy course. Anat Sci Educ 2013.
9. S B, E SK G. The development and evaluation of a computer-assisted teaching programme for intrapartum fetal monitoring. BJOG 2000(9).
10. Bekkers M, Simpson SA, Dunstan F, Hood K, Hare MEvans J, et al. Enhancing the quality of antibiotic prescribing in primary care: qualitative evaluation of a blended learning intervention. BMC Fam Pract 2010. 11: 11p.

11. Ben-Arye E, Lear A, Hermoni DMargalit RS. Promoting lifestyle self-awareness among the medical team by the use of an integrated teaching approach: a primary care experience. Journal of Alternative & Complementary Medicine 2007. 13(4): 461-469.
12. Bissell V, McKerlie RA, Kinane DFMcHugh S. Teaching periodontal pocket charting to dental students: a comparison of computer assisted learning and traditional tutorials. Brit Dent J 2003. 195(6): 333-336.
13. Blazer KR. Examining the Use of Distance-Mediated Case Conferencing for Case-Based Training in Clinical Cancer Genetics. ProQuest LLC, Ed.D. Dissertation, University of California, Los Angele 2010.
14. Bloomfield J, Roberts JWhile A. The effect of computer-assisted learning versus conventional teaching methods on the acquisition and retention of handwashing theory and skills in pre-qualification nursing students: a randomised controlled trial. Int J Nurs Stud 2010. 47(3): 287-294.
15. Bogacki RE, Best AAbbey LM. Equivalence study of a dental anatomy computer-assisted learning program. J Dent Educ 2004. 68(8): 867-871.
16. Bratt EVockell E. Using computers to teach basic facts in the nursing curriculum. J Nurs Educ 1986. 25(6): 247-251.
17. Bristol TJ. Hybrid learning in nursing education: Seat time plus feet time. Teaching and Learning in Nursing 2012. 7(4): 162-166.
18. Brocato CHeightman AJ. Sim Success: integrated training & simulation at the 2012 JEMS games. JEMS: Journal of Emergency Medical Services 2012. 37(8): 48-51.
19. Bryce E, Choi P, Landstrom MLoChang J. Using Online Delivery for Workplace Training in Healthcare. Journal of Distance Education 2008: 149-156.
20. Campagnolo DI, Stier KT, Sanchez W, Foye PMDeLisa JA. Spinal cord injury computer-assisted instruction for medical students. Am J Phys Med Rehab 2003. 82(4): 316-319.
21. Carr MM, Hewitt J, Scardamalia MReznick RK. Internet-based otolaryngology case discussions for medical students. J Otolaryngol 2002. 31(4): 197-201.
22. Carreras J. Molecular and cellular biology: An integrated course of biochemistry for medical students. Biochemical Education 1990. 18(4): 172-176.
23. Carreras J. Teaching of biochemistry at the faculty of medicine of the University of Barcelona: a new integrated curriculum. Biochemical Education 1997. 25(2): 81-82.
24. Casebeer L, Andolsek K, Abdolrasulnia M, Green J, Weissman NPryor E, et al. Evaluation of an Online Bioterrorism Continuing Medical Education Course. J Contin Educ Health 2006: 137-144.
25. Ceri P, Marangoni-Zuege MMorgan P. Process and Outcomes of Implementation of a Web-based Integrated Medication Dosage Calculation Learning and Assessment Program (eDose). Clinical Simulation in Nursing 2011. 7(6): e248.
26. HY CCH C. The learning effectiveness of nursing students using online testing as an assistant tool: a cluster randomized controlled trial. Nurs Educ Today 2012(3).
27. Chen B, Hirumi AZhang NJ. Investigating the Use of Advance Organizers as an Instructional Strategy for Web-Based Distance Education. Quarterly Review of Distance Education 2007: 223-231.
28. Chen KC, Glicksman JT, Haase P, Johnson M, Wilson TFung K. Introduction of a novel teaching paradigm for head and neck anatomy. J Otolaryngol Head Neck Surg 2010. 39(4): 349-355.
29. Chen SSChou P. The Implication of Integrated Training Program for Medical History Education. Biomed J 2014.
30. Chen YY, MUTUAL LEARNING THROUGH WEB-HYBRID TEACHING: AN INTERIM STEP TO DEVELOPING A FULLY ONLINE COURSE, in 2011 4TH INTERNATIONAL CONFERENCE OF EDUCATION, RESEARCH AND INNOVATION

(ICERI). 2011. p. 1424-1430.
31. Chew FSSmirniotopoulos JG. Educational efficacy of computer-assisted instruction with interactive videodisc in radiology. Invest Radiol 1993. 28(11): 1052-1058.
32. Chew FSStiles RS. Joseph E Whitley, MD, Award. Computer-assisted instruction with interactive videodisc versus textbook for teaching radiology. Acad Radiol 1994. 1(4): 326-331.
33. Chew FSLanier L. Learning radiology from interactive videodiscs: Bar-code book versus computer-assisted instruction. Acad Radiol 1995. 2(11): 1016-1020.
34. Chiu S, Cheng K, Sun T, Chang K, Tan TLin T, et al. The effectiveness of interactive computer assisted instruction compared to videotaped instruction for teaching nurses to assess neurological function of stroke patients: a randomized controlled trial. Int J Nurs Stud 2009. 46(12): 1548-1556.
35. Collins J, Dottl SLAlbanese MA. Teaching Radiology to Medical Students: An Integrated Approach. Acad Radiol 2002. 9(9): 1046-1053.
36. Dean PJ, Stahl MJ, Sylwester DLPeat JA. Effectiveness of Combined Delivery Modalities for Distance Learning and Resident Learning. Effectiveness of Combined Delivery Modalities for Distance Learning and Resident Learning 2001. 2(3): 247-254.
37. DeBate RD, Severson HH, Cragun DL, Gau JM, Merrell LKBleck JR, et al. Evaluation of a theory-driven e-learning intervention for future oral healthcare providers on secondary prevention of disordered eating behaviors. HEALTH EDUCATION RESEARCH 2013. 28(3): 472-487.
38. Delafuente JC, Araujo OELegg SM. Traditional lecture format compared to computer-assisted instruction in pharmacy calculations. Am J Pharm Educ 1998. 62(1): 62-66.
39. Delver H, Jackson W, Lee SPalacios M. FM POD: an evidence-based blended teaching skills program for rural preceptors. Fam Med 2014. 46(5): 369-377.
40. Dennis KK. The Effectiveness of an Online Fitness Course. ProQuest LLC, Ed.D. Dissertation, Illinois State University 2011.
41. Denny MHiggins A. The use of computer assisted technology to enhance student psychiatric nurses learning during a practice placement. Nurse Education in Practice 2003. 3(2): 80-88.
42. Desai N, Philpott-Howard J, Wade JCasewell M. Infection control training: evaluation of a computer-assisted learning package. J Hosp Infect 2000. 44(3): 193-199.
43. Dimeff LA, Woodcock EA, Harned MSBeadnell B. Can Dialectical Behavior Therapy Be Learned in Highly Structured Learning Environments? Results from a Randomized Controlled Dissemination Trial. Behavior Therapy 2011: 263-275.
44. Donabedian DDonabedian A. Effectiveness of computer-aided learning in community health nursing. Computers in Nursing 1993. 11(3): 101-112.
45. Dreher MA, The effect of instructor modeling of CAI as a computer-mediated lecture on student use of CAI, attitudes toward CAI, and achievement.. 1994, NORTHERN ILLINOIS UNIVERSITY. p. 186.
46. GJ D. A comparison of the effectiveness of computer-based learning courses among nursing staff. Journal for nurses in staff development 2008(2).
47. Dusenbury LA, Hansen WBGiles SM. Teacher Training in Norm Setting Approaches to Drug Education: A Pilot Study Comparing Standard and Video-Enhanced Methods. Journal of Drug Education 2003: 325-336.
48. Eryilmaz E, van der Pol J, Ryan T, Clark PMMary J. Enhancing Student Knowledge Acquisition from Online Learning Conversations. International Journal of Computer-Supported Collaborative Learning 2013: 113-144.
49. Fancovicova J, Prokop PUsak M. Web-Site as an Educational Tool in Biology Education: A Case of Nutrition Issue. Educational Sciences: Theory and Practice 2010: 907-921.

50. VD F, A BC L. Development and testing of a CD-ROM based tutorial for nursing students: getting ready for HIPAA. J Nurs Educ 2005(8).
51. Fernández Alemán JL, Carrillo De Gea JMRodríguez Mondéjar JJ. Effects of competitive computer-assisted learning versus conventional teaching methods on the acquisition and retention of knowledge in medical surgical nursing students. Nurs Educ Today 2011. 31(8): 866-871.
52. Finkelstein JLapshin O. Reducing depression stigma using a web-based program. Int J Med Inform 2007. 76(10): 726-734.
53. Ford GS, Mazzone MATaylor K. Effect of computer-assisted instruction versus traditional modes of instruction on student learning of musculoskeletal special tests. Journal of Physical Therapy Education 2005. 19(2): 22-30.
54. Francis B, Mauriello SM, Phillips C, Englebardt SGrayden SK. Assessment of Online Continuing Dental Education in North Carolina. J Contin Educ Health 2000: 76-84.
55. Garfunkel LC, Pisani AR, LeRoux PSiegel DM. Educating Residents in Behavioral Health Care and Collaboration: Comparison of Conventional and Integrated Training Models. Acad Med 2011. 86(2): 174-179.
56. Garrud P, Chapman IR, Gordon SAHerbert M. Non-verbal communication: evaluation of a computer-assisted learning package. Med Educ 1993. 27(6): 474-478.
57. Ghosh SPandya HV. Implementation of Integrated Learning Program in neurosciences during first year of traditional medical course: Perception of students and faculty. BMC Med Educ 2008. 8(44).
58. Gibbard LLSalajan F. A Novel Interactive Online Module in a Traditional Curriculum through a Blended Learning Approach. Electronic Journal of e-Learning 2009: 301-308.
59. Gilbert DAKolacz NG. Effectiveness of computer assisted instruction and small-group review in teaching clinical calculation. Computers in Nursing 1993. 11(2): 72-77.
60. Grigg PA, Stephans CDEaton KA. Evaluation of two computer assisted learning programs for dental nurses. J Dent Res 1998. 77(B): 831.
61. Hall LO, Soderstrom T, Ahlqvist JNilsson T. Collaborative Learning with Screen-Based Simulation in Health Care Education: An Empirical Study of Collaborative Patterns and Proficiency Development. Journal of Computer Assisted Learning 2011: 448-461.
62. Halverson AL, DaRosa DA, Borgstrom DC, Caropreso PR, Hughes TGHoyt DB, et al. Evaluation of a blended learning surgical skills course for rural surgeons. Am J Surg 2014. 208(1): 136-142.
63. SS HBL W. Teaching ergonomics to nursing facility managers using computer-based instruction. Journal for nurses in staff development 2006(5).
64. SS HBL W. A comparison of computer-based and instructor-led training for long-term care staff. J Contin Educ Nurs 2002(1).
65. SS HBL W. The effects of computer-based training on immediate and residual learning of nursing facility staff. J Contin Educ Nurs 2004(4).
66. SS HBL W. Is computer-based instruction an effective way to present fire safety training to long-term care staff? Journal for nurses in staff development 2003(3).
67. Harris Jr JM, Salasche SJHarris RB. Using the Internet to teach melanoma management guidelines to primary care physicians. J Eval Clin Pract 1999. 5(2): 199-211.
68. Hastings AM, Fraser RCMcKinley RK. Student perceptions of a new integrated course in clinical methods for medical undergraduates. Med Educ 2000. 34(2): 101-107.
69. Hemmati N, Omrani SHemmati N. A Comparison of Internet-Based Learning and Traditional Classroom Lecture to Learn CPR for Continuing Medical

Education. Turkish Online Journal of Distance Education 2013: 256-265.
70. Herriot AM, Bishop JA, Kelly M, Murphy MTruby H. Evaluation of a computer assisted instruction resource in nursing education. Nurs Educ Today 2003. 23(7): 537-545.
71. Hodge JE, The effect of math anxiety, math self-efficacy, and computer-assisted instruction on the ability of undergraduate nursing students to calculate drug dosages.. 2002, West Virginia University. p. 106.
72. Howerton WJ, Platin E, Ludlow JTyndall DA. The influence of computer-assisted instruction on acquiring early skills in intraoral radiography. J Dent Educ 2002. 66(10): 1154-1158.
73. Hulsman RL, Ros WJ, Winnubst JABensing JM. The effectiveness of a computer-assisted instruction programme on communication skills of medical specialists in oncology. Med Educ 2002. 36(2): 125.
74. Ilic D, Bin NR, Glasziou P, Tilson JKVillanueva E. Implementation of a blended learning approach to teaching evidence based practice: a protocol for a mixed methods study. BMC Med Educ 2013. 13: 170.
75. Jacoby CG, Smith WLAlbanese MA. An evaluation of computer-assisted instruction in radiology. AJR Am J Roentgenol 1984. 143(3): 675-677.
76. Jahanbani J, Mirlashari JFahimi O. The effectiveness of an oral pathology computer-assisted learning program for dental students. Journal of Dentistry (17283426) 2010. 10: 57-60.
77. Jamkar A, Yemul VSingh G. Integrated teaching programme with student-centred case-based learning. Med Educ 2006. 40(5): 466-467.
78. Jang KS, Hwang SY, Park SJ, Kim YMKim MJ. Effects of a Web-based teaching method on undergraduate nursing students' learning of electrocardiography. J Nurs Educ 2005. 44(1): 35-39.
79. Jian W, Qi ZXin D. Preliminary research in the application of integrated learning and teacher-centredness in undergraduate education in China. Med Teach 2011. 33(4): e178-e185.
80. Jonas DBurns B. The transition to blended e-learning. Changing the focus of educational delivery in children’s pain management. Nurse Education in Practice 2010. 10(1): 1-7.
81. Kachroo M. Effect of computer-assisted teaching programme on the knowledge of nursing personnel about pre-term labour and low birth weight. Nurs J India 2012. 103(2): 65-68.
82. Kandasamy TFung K. Interactive Internet-based cases for undergraduate otolaryngology education. Otolaryngol Head Neck Surg 2009. 140(3): 398-402.
83. Koch J, Andrew S, Salamonson Y, Everett BDavidson PM. Nursing students' perception of a web-based intervention to support learning. Nurs Educ Today 2010. 30(6): 584-590.
84. Laraia BA, Dodds JM, Benjamin S, Jones SJCarbone ET. Can Distance Education Prepare Future Public Health Nutritionists? A Case Study. J Nutr Educ Behav 2008: 34-38.
85. VR L, A U, F HRM L. A preliminary study in using virtual reality to train dental students. J Dent Educ 2004(3).
86. Lechner SK, Lechner KMThomas GA. Evaluation of a computer-aided learning program in removable partial denture framework designing. J Prosthodont 1999. 8(2): 100-105.
87. Lee A, Joynt GM, Ho AM, Gin THazlett CB. Effect of an integrated teaching intervention on clinical decision analysis: a randomized, controlled study of undergraduate medical students. Med Teach 2007. 29(2-3): 231-236.

88. Lee RLT, Wong TKS, Al-Gasseer N, Wu CST, Chan SSSKo SKK, et al. Evaluating the efficacy of an integrated curriculum on adolescent health and development for pre-service nursing education in Hong Kong. Nurs Educ Today 2006. 26(4): 286-297.
89. Leong C, Shakespeare TP, Mukherjee R, Back MF, Lee KLu JJ, et al. Effect of an integrated continuing medical education (CME) and quality improvement (QI) program on radiation oncologist (RO) clinical practice. International Journal of Radiation Oncology*Biology*Physics 2004. 60(1, Supplement): S555-S556.
90. Leung KH, Pluye P, Grad RWeston C. A Reflective Learning Framework to Evaluate CME Effects on Practice Reflection. J Contin Educ Health 2010: 78-88.
91. Lewin LO, Singh M, Bateman BLGlover PB. Improving education in primary care: development of an online curriculum using the blended learning model. BMC Med Educ 2009. 9: 33.
92. Lieberman G, Abramson R, Volkan KMcArdle PJ. Tutor versus Computer: A Prospective Comparison of Interactive Tutorial and Computer-Assisted Instruction in Radiology Education. Acad Radiol 2002. 9(1): 40-49.
93. Li-Ling H. Qualitative Assessment of a Blended Learning Intervention in an Undergraduate Nursing Course. Journal of Nursing Research (Lippincott Williams & Wilkins) 2012. 20(4): 291-298.
94. TJ L, JM C, LA JFJ W. Effectiveness of computer-aided removable partial denture design. Journal of prosthodontics 1997(2).
95. Liu W, Rong JLiu C. Using evidence-integrated e-learning to enhance case management continuing education for psychiatric nurses: A randomised controlled trial with follow-up. Nurs Educ Today 2014. 34(11): 1361-1367.
96. Long AF, Mercer PE, Stephens CDGrigg P. The evaluation of three computer-assisted learning packages for general dental practitioners. Br Dent J 1994. 177(11-12): 410-415.
97. Longmuir KJ. Interactive computer-assisted instruction in acid-base physiology for mobile computer platforms. Adv Physiol Educ 2014. 38(1): 34-41.
98. Longo A, Hensley BSchilling S. Striving for Excellence in Central Venous Catheter Care: Applying a Blended Learning Approach to Nursing Staff Education on CVC Bundle Care Practices Aimed at Reducing Catheter-Associated Blood Stream Infection. Journal of Pediatric Nursing 2008. 23(2): e18-e19.
99. Lu FLemonde M. A Comparison of Online versus Face-to-Face Teaching Delivery in Statistics Instruction for Undergraduate Health Science Students. Adv Health Sci Educ 2013: 963-973.
100. Lynch TG, Steele DJ, Johnson Palensky JE, Lacy NLDuffy SW. Learning preferences, computer attitudes, and test performance with computer-aided instruction. The American Journal of Surgery 2001. 181(4): 368-371.
101. Mackenburg-Mohn MD, Enhancing critical thinking in baccalaureate nursing students through the use of computer assisted learning activities.. 2006, Capella University. p. 183.
102. Madorin SIwasiw C. The effects of computer-assisted instruction on the self-efficacy of baccalaureate nursing students. J Nurs Educ 1999. 38(6): 282-285.
103. Majagi SI. Introduction of Computer Assisted Learning and Its Comparison With Traditional Practicals in Pharmacology for Under Graduates. Indian J Pharmacol 2013. 451: S86-S87.
104. Maloney S, Storr M, Paynter S, Morgan PIlic D. Investigating the Efficacy of Practical Skill Teaching: A Pilot-Study Comparing Three Educational Methods. Adv Health Sci Educ 2013: 71-80.
105. Maresca C, Barrero C, Duggan D, Platin E, Rivera EHannum W, et al. Utilization of blended learning to teach preclinical endodontics. J Dent Educ 2014. 78(8): 1194-1204.
106. McCracken GI, Nunn JH, Hobson RS, Stephenson JJJepson N. Evaluation of a computer-assisted learning package on the management of traumatised

incisors by general dental practitioners. ENDODONTICS & DENTAL TRAUMATOLOGY 2000. 16(1): 40-42.
107. McKeough DM, Mattern-Baxter KBarakatt E. Effectiveness of a computer-aided neuroanatomy program for entry-level physical therapy students: anatomy and clinical examination of the dorsal column- medial lemniscal system. Journal of Allied Health 2010. 39(3): 156-164.
108. McMullan M, Jones RLea S. The effect of an interactive e-drug calculations package on nursing students' drug calculation ability and self-efficacy. Int J Med Inform 2011. 80(6): 421-430.
109. McNulty JA, Sonntag BSinacore JM. Evaluation of computer-aided instruction in a gross anatomy course: a six-year study. Anat Sci Educ 2009. 2(1): 2-8.
110. Meckfessel S, Stuhmer C, Bormann KH, Kupka T, Behrends MMatthies H, et al. Introduction of e-learning in dental radiology reveals significantly improved results in final examination. J Cranio Maxill Surg 2011. 39(1): 40-48.
111. Miedzybrodzka Z, Hamilton NM, Gregory H, Milner B, Frade ISinclair T, et al. Teaching undergraduates about familial breast cancer: comparison of a computer assisted learning (CAL) package with a traditional tutorial approach. Eur J Hum Genet 2001. 9(12): 953-956.
112. Mileman PA, van den Hout WBSanderink G. Randomized controlled trial of a computer-assisted learning program to improve caries detection from bitewing radiographs. Dentomaxillofac Rad 2003. 32(2): 116-123.
113. Mitchell BW, The influence of computer-assisted instruction on the rate of universal precautions related behaviors.. 1995, UNIVERSITY OF ALABAMA AT BIRMINGHAM. p. 94.
114. ML M. Hypertext computer-assisted instruction for geriatric physical therapists. PHYS. OCCUP. THER. GERIATR. 1991(2).
115. Moss SH, Redfern PH, Pouton CW, Brown KNGilbert M. P298 computer aided learning programs in pharmacy and pharmacology. Eur J Pharm Sci 1994. 2(1–2): 193.
116. Murphy-Ende K, The relationship of self-directed learning, self-efficacy, and health value in young women with cancer using a computer health education program.. 1996, UNIVERSITY OF WISCONSIN - MADISON. p. 218.
117. Nerlich SM. Using Computer-Assisted Instruction (CAI) to Enhance Nursing Education and Practice. Aust Crit Care 1995. 8(2): 30-31.
118. Pahinis K, Stokes CW, Walsh TFCannavina G. Evaluating a blended-learning course taught to different groups of learners in a dental school. J Dent Educ 2007. 71(2): 269-278.
119. Panikkar J, Draycott TCook J. The evaluation of computer-aided learning in medicine. Postgrad Med J 1998. 74(878): 706-708.
120. Perciful EG. The relationship between planned change and successful implementation of computer assisted instruction. Computers in Nursing 1992. 10(2): 85-90.
121. Probst H, Eddy D, Doughty JHodgson D. Integrating E-Learning into Postgraduate Radiotherapy and Oncology Education: A Case Study. E-Learning 2009: 363-371.
122. Richardson D. Student perceptions and learning outcomes of computer-assisted versus traditional instruction in physiology. Am J Physiol 1997. 273(6 Pt 3): S55-S58.
123. Ricks C, Ratnapalan S, Jain STait G. Evaluating computer-assisted learning for common pediatric emergency procedures. Pediatr Emerg Care 2008. 24(5): 284-286.
124. Rigby L, Wilson I, Baker J, Walton T, Price ODunne K, et al. The development and evaluation of a ‘blended’ enquiry based learning model for mental health nursing students: “making your experience count”. Nurs Educ Today 2012. 32(3): 303-308.

125. Riley WAnderson P. Randomized Study of the Impact of Cooperative Learning: Distance Education in Public Health. Quarterly Review of Distance Education 2006: 129-144.
126. Robson J. Web-based learning strategies in combination with published guidelines to change practice of primary care professionals. Brit J Gen Pract 2009. 59(559): 104-109.
127. Rodriguez EL, The use of blended learning to facilitate critical thinking in entry level occupational therapy students.. 2009, Capella University. p. 144.
128. Roh YSKim SS. The Effect of Computer-Based Resuscitation Simulation on Nursing Students' Performance, Self-Efficacy, Post-Code Stress, and Satisfaction. Res Theor Nurs Pract 2014. 28(2): 127-139.
129. Rohlman DS, Eckerman DA, Ammerman TA, Fercho HL, Lundeen CABlomquist C, et al. Quizzing and Feedback in Computer-Based and Book-Based Training for Workplace Safety and Health. Journal of Organizational Behavior Management 2005: 1-26.
130. S R, FC Sde Andrade CR F. Computer game-based and traditional learning method: a comparison regarding students' knowledge retention. BMC Med Educ 2013.
131. Rosenberg H, Posluns J, Tenenbaum HC, Tompson BLocker D. Evaluation of computer-aided learning in orthodontics. Am J Orthod Dentofac 2010. 138(4): 410-419.
132. Russell KM, Miller AMCzerwinska J. Epidemiology for community health nursing: an interactive computer assisted instruction program. Computers in Nursing 1994. 12(2): 98-105.
133. Salamonson YLantz J. Factors influencing nursing students' preference for a hybrid format delivery in a pathophysiology course. Nurs Educ Today 2005. 25(1): 9-16.
134. Schaefer BJ, Strategies nurse educators use to integrate computer-assisted instruction into their courses to teach clinical decision-making.. 2005, West Virginia University. p. 503.
135. S S, D J, R P, K W, C PT K, et al. Evaluation of an online Diabetes Needs Assessment Tool (DNAT) for health professionals: a randomised controlled trial. Trials 2009.
136. Shaw T, Long A, Chopra SKerfoot BP. Impact on Clinical Behavior of Face-to-Face Continuing Medical Education Blended with Online Spaced Education: A Randomized Controlled Trial. J Contin Educ Health 2011. 31(2): 103-108.
137. Sherriff K, Burston SWallis M. Effectiveness of a computer based medication calculation education and testing programme for nurses. Nurs Educ Today 2012. 32(1): 46-51.
138. Shumaker JPenny M. Understanding the Impact of Using Mass Media as a Pedagogical Tool for Nutrition Education of Healthcare Workers in the Community College Classroom. ProQuest LLC 2011.
139. So HBrush TA. Student perceptions of collaborative learning, social presence and satisfaction in a blended learning environment: Relationships and critical factors. Comput Educ 2008. 51(1): 318-336.
140. Stark CM, Graham-Kiefer ML, Devine CM, Dollahite JSOlson CM. Online Course Increases Nutrition Professionals' Knowledge, Skills, and Self-Efficacy in Using an Ecological Approach to Prevent Childhood Obesity. J Nutr Educ Behav 2011: 316-322.
141. RG S, YP W, CC CCD J. Evaluation of child health matters: a web-based tutorial to enhance school nurses' communications with families about weight-related health. J Sch Nurs 2013(2).

142. Stegeman CA. The Effect of a Multimedia Learning Environment on the Knowledge, Attitude, Confidence, and Skill of Dental Hygiene Students. ProQuest LLC, Ed.D. Dissertation, University of Cincinnati 2011.
143. Stephens MHennefer D. Internationalising the nursing curriculum using a Community of Inquiry Framework and blended learning. Nurse Education in Practice 2013. 13(3): 170-175.
144. Stokes CW, Cannavina CCannavina G. The state of readiness of student health professionals for web-based learning environments. Health Inform J 2004. 10(3): 195-204.
145. Tarling M. The development and evaluation of a new blended learning ambulatory surgery nursing course. Journal of One-Day Surgery 2012. 22(1): 17-19.
146. Tashiro J, Setoyama Y, Hirabayashi Y, Nagamatsu YOmori J. Process and evaluation of the first year Web-based service learning course [Japanese]. Bulletin of St. Luke's College of Nursing 2011(37): 25-30.
147. Thomas RLAllen RM. Use of computer-assisted learning module to achieve ACGME competencies in orthopaedic foot and ankle surgery. Foot Ankle Int 2003. 24(12): 938-941.
148. JS T, B L, S SF K. Knowledge of quality performance measures associated with endoscopy among gastroenterology trainees and the impact of a web-based intervention. Gastrointest Endosc 2012(1).
149. Tsai S, Tsai W, Chai S, Sung W, Doong JFung C. Evaluation of computer-assisted multimedia instruction in intravenous injection. Int J Nurs Stud 2004. 41(2): 191-198.
150. Van De Mortel TF, Trigger R, Ahern CBird J. Evaluating a community-engaged vertically integrated teaching and learning pilot project. Education for Primary Care 2013. 24(3): 165-172.
151. Vollmar HC, Mayer H, Ostermann T, Butzlaff ME, Sandars JEWilm S, et al. Knowledge transfer for the management of dementia: a cluster randomised trial of blended learning in general practice. Implement Sci 2010. 5: 1.
152. BL WSS H. Can nursing facility staff with minimal education be successfully trained with computer-based training? Nurs Educ Today 2004(4).
153. Walsh CM, Rose DN, Dubrowski A, Ling SC, Grierson LBackstein D, et al. Learning in the Simulated Setting: A Comparison of Expert-, Peer-, and Computer-Assisted Learning. Acad Med 2011. 86S(10): S13-S17.
154. Walsh K. Evaluation of a blended learning model in geriatric medicine: The need to delve more deeply. Australas J Ageing 2013. 32(4): 249.
155. Waugh RA, Mayer JW, Ewy GA, Felner JM, Issenberg BSGessner IH, et al. Multimedia computer-assisted instruction in cardiology. Arch Intern Med 1995. 155(2): 197-203.
156. Welbury RR, Hobson RS, Stephenson JJJepson N. Evaluation of a computer-assisted learning programme on the oro-facial signs of child physical abuse (nonaccidental injury) by general dental practitioners. Brit Dent J 2001. 190(12): 668-670.
157. Wilson TMires G. Education. Teacher versus the computer for instruction: a study. British Journal of Midwifery 1998. 6(10): 655-658.
158. Woodall B. The development and evaluation of a new blended learning ambulatory surgery nursing course: JODS 22.1. Journal of One-Day Surgery 2012. 22(2): 51.
159. Xakellis GCGjerde C. Evaluation by second-year medical students of their computer-aided instruction. Acad Med 1990. 65(1): 23-26.
160. Yeung JC, Fung KWilson TD. Prospective evaluation of a web-based three-dimensional cranial nerve simulation. J Otolaryngol Head Neck Surg 2012. 41(6): 426-436.

161. Yoshida N, Aso T, Asaga T, Okawa Y, Sakamaki HMasumoto T, et al. Introduction and evaluation of computer-assisted education in an undergraduate dental hygiene course. Int J Dent Hyg 2012. 10(1): 61-66.
162. Zebrack JR, Mitchell JL, Davids SLSimpson DE. Web-based curriculum - A practical and effective strategy for teaching women's health. J Gen Intern Med 2005. 20(1): 68-74.
163. Zolfaghari M, Negarandeh RAhmadi F. The Evaluation of a Blended E-learning Program for Nursing and Midwifery Students in Tehran University of Medical Sciences [Farsi]. Iranian Journal of Medical Education 2011. 10(4): 1-12.

Section 2: articles excluded due to insufficient data(n=6)

1. Bains M, Reynolds PA, McDonald FSherriff M. Effectiveness and acceptability of face-to-face, blended and e-learning: a randomised trial of orthodontic undergraduates. Eur J Dent Educ 2011. 15(2): 110-117.
2. RD F, C HPJ N. A comparative assessment of interactive videodisc instruction. Computers in nursing 1993(5).
3. Goodie JL, Williams PM, Kurzweil DMarcellas KB. Can blended classroom and distributed learning approaches be used to teach medical students how to initiate behavior change counseling during a clinical clerkship? J Clin Psychol Med Settings 2011. 18(4): 353-360.
4. Grasl MC, Pokieser P, Gleiss A, Brandstaetter J, Sigmund TErovic BM, et al. A new blended learning concept for medical students in otolaryngology. Arch Otolaryngol Head Neck Surg 2012. 138(4): 358-366.
5. Perkins GD, Kimani PK, Bullock I, Clutton-Brock T, Davies RPGale M, et al. Improving the efficiency of advanced life support training: a randomized, controlled trial. Ann Intern Med 2012. 157(1): 19-28.
6. Qayumi AK, Kurihara Y, Imai M, Pachev G, Seo HHoshino Y, et al. Comparison of computer-assisted instruction (CAI) versus traditional textbook methods for training in abdominal examination (Japanese experience). Med Educ 2004. 38(10): 1080-1088.

E-Table 3. Description of included publications

Section 1. Studies comparing blended learning with no intervention

Study	Design RCT/NRS)	Country	Participants no.(B/N)[a]; type	Topic	Study intervention(component or features)	Modality or technology	Intervention duration	Exercises	Interactivity	Discussion	Delay between posttest and course	Assessment(question type)	Conflict of interest	Funding from company	Quality score
Flys,2012	Pre-posttest 1-group; NRS	Panama, Nicaragua, Dominican Republic, and Guatemala	225; Doctors, nurses, psychologists, health administrators, etc.	Principles of HIV care and health systems	Online+on-site +projects	Moodle; on-site workshops	150 hours (10 weeks)	Present(cases, self-assessment)	High(essays, group work)	Present	No delay	Subjective (MCQ ,essays)	No	No	4
Puri,2010	Pre-posttest, 2 groups ; NRS	Indiana	350/102; Dietetic students	Communication and counseling skills	CAI+print resources	Internet-based site	1 week	Present(cases)	Low	Absent	No delay	Objective(cannot tell)	No	No	6
Karaksha,2011	Posttest, 2 groups ;RCT	Australia	23/17;Pharmacy students	Pharmacology	CAL+lecture	CD, iSpring Pro 4.3.0.	24 hours	Present(quiz,assessment)	Low	Absent	No delay	Objective(MCQ)	No	No	4
Buchowski,2002	Pre-posttest, 1 group; NRS	USA	80/78;Medical students	Nutritional Anemias and the Diabetes and Weight Management	CAI+traditional	Aberrations in Glucose Metabolism modules	1 semester	Present(cases, self-assessment)	High(group work)	Absent	3 months	Objective(cannot tell)	No	Yes	3
Wallen,2010	Pre-posttest 1-group; NRS	USA	127;-nurses	Basic genetics	Web-based+face-toface	self-paced learning modules	<1 semester	Absent	Low	Absent	No delay	Objective(problem-based questions)	No	No	3
Weaver,2014(a)	Pre-posttes	USA	60; Health	Health policy	e-learning+traditional	technology-driven	12 mont	Present(cases, self-	High(team-baased	Pre sen	No dela	Subjective(analysis,interpretation,inference,	No	No	3

	t 1-group; NRS		students			combined platform, blackboard-uploaded virtual interactive lectures	hs	assessment)	assignment)	t	y	evaluation)			
Weaver,2014(b)	Pre-posttest 1-group; NRS	USA	60; Health students	Health policy	e-learning+traditional	technology-driven combined platform, blackboard-uploaded virtual interactive lectures	12 months	Present	High	Present	No delay	Subjective(analysis,interpretation,inference, evaluation)	No	No	3
Riesen,2012	Pre-posttest 1-group; NRS	Canada	60;Health graduates	Interprofessional competencies	virtual face-to-face +traditional face-to-face+online	real-life simulation, virtual simulation, virtual debriefing and a didactic learning component. Web.Alive	2 days	Absent	High(discussion group)	Present	No delay	Subjective(self-report)	No	Yes	3
Cho,2014(a)	Pre-posttest 1-group; NRS	South Korea	45; Nurses	Research ethic	Web-based online instruction + a off-line instruction(review of the core contents on the online program, case analysis, small group discussion and miscellaneous activities)	develope Analysis, Design, Development, Implementation, and Evaluation (ADDIE) model	30 hours	Present(cases	High(group discussion)	Present	No delay	Objective(cannot tell)	No	No	3
Cho,2014(b)	Pre-posttest 1-group; NRS	South Korea	69;Nursing students	Research ethic	Web-based online instruction + a off-line instruction(review of the core contents on the online program, case analysis, small group discussion and miscellaneous activities)	Analysis, Design, Development, Implementation, and Evaluation (ADDIE) model	30 hours	Present(cases	High(group discussion)	Present	No delay	Objective(cannot tell)	No	No	3
Pereira,2008(a)	Pre-posttest 1-group;	Canada	14;Family medicine resident	Palliative care	Web-based learning+face-toface workshop	asynchronous discussion forums, a	8 weeks	Absent	High(group-based discussion)	Present	No delay	Objective(MCQ)	No	No	4

	NRS		s			live audio and text-based online synchronous session(Centra); online modules (Macromedia Breeze)								
Pereira,2008(b)	Pre-posttest 1-group; NRS	Canada	16;Family medicine residents	Palliative care	Web-based learning+face-toface workshop	asynchronous discussion forums a live audio-and text-based online synchronous session(Centra); online modules (Macromedia Breeze)	8 weeks	Absent	High(group-based discussion)	Present	No delay	Objective(MCQ)	No	No	4
Karamizadeh,2011 (a)	Pre-posttest 1-group; NRS	Iran	10;Medical students	Medical training	E-learning+class session	Multimedia compact disk	4 weeks	Absent	High(problem solving session)	Absent	2-4 weeks delay	Objective(cannot tell)	No	No	3
Karamizadeh,2011 (b)	Pre-posttest 1-group; NRS	Iran	40;Extern	Medical training	E-learning+class session	Multimedia compact disk	4 weeks	Absent	High(problem solving session)	Absent	2-4 weeks delay	Objective(cannot tell)	No	No	3
Karamizadeh,2011 (c)	Pre-posttest 1-group; NRS	Iran	19;Intern	Medical training	E-learning+class session	Multimedia compact disk	4 weeks	Absent	High(problem solving session)	Absent	2-4 weeks delay	Objective(cannot tell)	No	No	3
Karamizadeh,2011 (d)	Pre-posttest 1-group; NRS	Iran	38; Resident	Medical training	E-learning+class session	Multimedia compact disk	4 weeks	Absent	High(problem solving session)	Absent	2-4 weeks delay	Objective(cannot tell)	No	No	3
Karamizadeh,2011	Pre-	Iran	6;Assista	Medical	E-learning+class	Multimedia	4	Absent	High(probl	Abs	2-4	Objective(cannot tell)	No	No	3

(e)[1][1][21][1]	posttest 1-group; NRS		nt professor	training	session	compact disk	weeks		em solving session)	ent	weeks delay				
Chandler,2008	Pre-posttest 1-group; NRS	USA	817;Public health workers	Emergency preparedness	Web-based+on-the-job+face-to-face; downloadable homework assignment	Website; downloadable template	2 days	Absent	High(required for response)	Present	No delay	Objective(MCQ)	No	No	3
Baumlin 2006	Posttest, 2 group; RCT	USA	40/50; Clinical medical students	Lung cancer	Web tutorial +traditional	Internet tutor, repetition	1-4weeks	Present(case)	Low(case)	Present	No delay	Objective(cannot tell)	No	No	3
Cragun, 2005	Pre-posttest, 1 group; NRS	USA	39/15Nursing students	Genetics	Web tutorial +face-to-face lecture	Web tutorial	1 day	Present(case-based problem)	Low	Absent	No delay	Objective(MCQ, true or false question)	No	No	3

Section 2.Studies comparing blended learning with non-blended learning

Study	Design RCT/NRS)	Country	Comparison intervention	Participants no.(B/N[a]); type	Topic	Study intervention(component or features)	Modality or technology	Duration	Exercises	Interactivity	Discussion	Delay between posttest and course	Assessment(question type)	Conflict of interest;	Source of support	Quality score
Kulier, 2012	Pre-posttest 2 groups ;RCT	7 LMICs (Argentina, Brazil, Democratic Republic of the	Traditional teaching	123/81; Postgraduate trainees	Reproductive Health	E-learning+F2F	recorded video, specialist database	8 weeks	Present(questions, assignments)	High(Feedback on assignments)	Absent	4 weeks	Objective(MCQ)	No	No	6

		Congo, India, Philippines, South Africa, Thailand).														
Kavadella,2012	Pre-posttest 2 group; RCTs	Greece	Conventional face to face methodology	24/22; Undergraduate	Oral radiology	F2F + online	E-learning platform Web-based tools include self-graded tests and quizzes, online discussion groups	0.5 year	Present(self-graded tests and quizzes)	High(self-graded tests and quizzes, online discussion groups)	Present	No delay	Objective(dichotomal :yes/no)	No	No	4
Lancaster,2011	Pre-posttest, 1 group; NRS	USA	Traditional	97; Second professional year students	Pharmacy curriculum	online self-directed study +in-class active learning	Blackboard online hosting service	1 semester	Present(quiz	High(quiz, group discussion)	Present	No delay	Objective(Choice question)	No	No	3
Sowan,2013	Posttest, 2 groups; RCT	Jordan	Traditional format	105/105; undergraduate nursing students	Scientific research in nursing	Web-based+interactive F2F	Blackboard and Tegrity systems	1 semester	Present(questions, assignments)	High (questions, assignments)	Present	No delay	Objective(open-ended questions)	No	No	5
Makhdoom,2013	Posttest, 2 groups ;RCT	Saudi Arabia	face-to-face	60/61; Medical students	Family medicine course	E-learning+F2F	Electronic course management system	10 weeks	Absent	High(interact with tutors)	Present	No delay	Objective(MCQ)	No	No	5
Lancaster,2012	Posttest, 2 groups ;RCT	USA	Traditional in-class	29/23; Graduate	Pharmacotherapeutics course	Oline+F2F	Griffin Lapel Microphone, Articulate Presenter '09, electronic Blackboardhosting website	1 year	Present(assignment, question and answer sessions)	High (assignment, question and answer session ,question and answer sessions)	Present	No delay	Objective(Cannot tell)	No	No	4
Dankbaar,2014(a)	Posttest, 2 groups ;NRS	Netherlands	Traditional course	31/16; Nurse in postgraduate	Acute and intensive care	Online material+F2F lecture	Web lectures	11 days	Present (examples and exercises)	Low(examples and exercises with feedback)	Absent	No delay	Objective(MCQ)	No	No	5
Dankbaar,2014(b)[2][2][22][2]	Posttest, 2 groups ;NRS	Netherlands	Traditional course	31/16; Nurse in postgraduate	Acute and intensive care	Online material+F2F lecture	Web lectures	11 days	Present (examples and exercises)	Low(examples and exercises with feedback)	Absent	No delay	Objective(MCQ)	No	No	5

Dankbaar,2014(c)	Posttest, 2 groups;NRS	Netherlands	Traditional course	31/16; Nurse in postgraduate	Acute and intensive care	Online material+F2F lecture	Web lectures	11 days	Present (examples and exercises)	Low(examples and exercises with feedback)	Absent	No delay	Objective(MCQ)	No	No	5
Mangione,1991(a)	Pre-posttest, 2 groups;RCT	USA	Computer-assisted instruction	13/9; Medical students	Cardiac auscultation	Self-schedule CAI + small-group seminar	HEARTLAB platform,	12 weeks	Absent	Low	Absent	No delay	Objective(choice question)	No	No	4
Mangione,1991(b)	Pre-posttest, 2 groups;RCT	USA	Tutorial instruction	13/13; Medical students	Cardiac auscultation	Self-schedule CAI + small-group seminar	HEARTLAB platform,	12 weeks	Absent	Low	Absent	No delay	Objective(choice question)	No	No	4
Shomaker, 2002(a)	Pre-osttest, 2 groups; RCT	*USA*	traditional	24/24;medical students	parasitology	computer program + lectures	interactive text	2 weeks	Present(questions)	Low(questions)	Absent	No delay	Objective(MCQ or slides)	No	No	5
Shomaker, 2002(b)	Pre-posttest, 2 groups; RCT	*USA*	e-learning	24/17; medical students	parasitology		interactive text	2 weeks	Present(questions)	Low(questions)	Absent	No delay	Objective(MCQ or slides)	No	No	5
Stewart,2013	Posttest, 2 groups; RCT	Australia	standard teaching	34/37; Medical students	Newborn	Online module+standard programme	PENSKE Baby Check Learning module	8 weeks	Absent	Low	Absent	No delay	Objective(Cannot tell)	No	No	4
Mahnken,2011(a)	Pre-posttest, 2 groups; RCT	Germany	Traditional learning	32/32; Medical students	Radiology	E-learning+internship(F2F)	Electronic cases	1 week	Present (cases and expert feedback, question-and-answer	High (cases and expert feedback, question-and-answer)	Absent	No delay	Objective(Cannot tell)	No	No	4
Mahnken,2011(b)	Pre-posttest, 2 groups; RCT	Germany	Traditional learning	32/32; Medical students	Radiology	E-learning+internship(F2F)	Electronic cases	1 week	Present (cases and expert feedback, question-and-answer	High (cases and expert feedback, question-and-answer)	Absent	No delay	Objective(Cannot tell)	No	No	4
Sung, 2008	Pre-posttest, 2 groups;NRS	Korea	Face to face instruction	24/26; Nurses	Medical administration	Web-based matirilas +_face-to-face instruction	Web-based e-learning program	10 months	Present(quizzes with feedback, clinical cases)	High (quizzes with feedback, clinical cases, active interaction between tutors and students)	Absent	No delay	Objective(Cannot tell)	No	No	5
Woltering,2009	Posttes	Germa	traditiona	74/71;	Model	Online	multimedia	2	Present(questio	High(online	Pre	No	Objective(MCQ)	No	No	6

	t, 2 groups ;NRS	ny	l PBL	Medical students	Curriculum Medicine	learning+students' Meeting+tutored final session	case vignette, Group-Wiki, The virtual clinical order entry system, bulletin board	weeks	ns, cases)	collaboration including comments of the tutor)	sent	delay				
Karaksha,2011(a)	Posttest, 2 groups ; RCT	Australia	CAI	23/22; Pharmacy students	Pharmacology	Lecture+CAI	iSpring Pro 4.3.0, Blackboard, CD.	24H	Present (quiz, questions,multiple choice)	High(quiz, multiple choice essay questions)	Absent	No delay	Objective(MCQ)	No	No	5
Karaksha,2011(b)	Posttest, 2 groups ; RCT	Australia	Lecture	23/13; Pharmacy students	Pharmacology	Lecture+CAI	iSpring Pro 4.3.0, Blackboard, CD.	24H	Present (quiz, questions,multiple choice)	High(quiz, multiple choice essay questions)	Absent	No delay	Objective(MCQ)	No	No	5
Lowe,2001(a)	Posttest, 2 groups ;NRS	UK	lecture and seminar	39/46; Undergraduate dental students	Index of Orthodontic treatment need	CAL programme+seminar	Internet web-authoring package	1 week	Present(self-assessment)	High(multimedia design with interactive comment)	Present	No delay	Objective(cases)	No	No	5
Lowe,2001(b)	Posttest, 2 groups ;NRS	UK	lecture and seminar	39/46; Undergraduate dental students	Index of Orthodontic treatment need	CAL programme+seminar	Internet web-authoring package	1 week	Present (self-assessment)	High(multimedia design with interactive comment)	Present	No delay	Objective(cases)	No	No	5
**Hilger, 1996**	Pre-posttest, 2 groups ; RCT	*USA*	traditional	45/32;medical students	Streptococcal Pharyngitis	CAI program +clerkship	Online tutorial, case simulation	4 weeks	Present (case simulation,self-assessment)	High(discussion with feedback)	Present	No delay	Objective(MCQ. True or false)	No	No	5
Ilic,2013[3][3][23][3]	Posttest, 2 groups ;NRS	Australia	Didactic learning	34/27; Graduate medical students	Evidence based practice (EBP)	Tutorial sessions+web-site learning	Monash University library website	1 day	Present (patient-basedPresentation)	High(group work, patient-based Presentation)	Present	No delay	Objective(MCQ)	Yes	No	5
Daunt, 2013(a)	posttest, 2 groups ;NRS	*UK*	traditional	162/168;medical students	geriatric medicine	CAL package + traditional teaching	Xerte open access platform, Storyboards	4 weeks	Present(case,	High(case, interactive session)	Present	No delay	Objective(true or false, choice question, extended matching question)	No	No	3
Daunt, 2013(b)	posttest, 2 groups ;NRS	*UK*	traditional	92/67;medical students	geriatric medicine	CAL package + traditional teaching	Xerte open access platform, Storyboards	8 weeks	Absent	Low	absent	No delay	Objective(true or false, choice question, extended matching question)	No	No	3
Morales,2012[4][4][24][4]	Posttest, 2 groups	Spain	documents and books	22/22; Physiotherapy second-	Physiotherapy degree course	on-campus training+ website	ECOFISIO website	1 semester	Present(self-assessment)	Low(self-assessment)	Absent	No delay	Objective(MCQ)	No	No	5

	; RCT			year degree students		training										
Raupach,2010	Pre-posttest, 2 groups ; RCT	Germany	Traditional learning	40/34; Medical students	Cardio-respiratory curriculum	Online module+traditional curriculum	web-based learning management system	6 weeks	Present(test with feedback)	High(test with feedback)	Present	No delay	Objective(MCQ)	No	No	5
Carbonaro,2008	Pre-posttest, 2 groups ; RCT	Canada	face to face	22/22;Student	Health science program	E-learning+F2F interprofessional team course		5 weeks	Present(giving/ receiving feedback, consensus decision-making)	High(giving/ receiving feedback, consensus decision-making, Group discussions, problem solving)	Present	No delay	Subjective(cannot tell)	No	No	5
Pereira,2007	Posttest, 2 groups ;NRS	Spain	Traditional teaching	65/65;Students	Human anatomy	Online learning+seminars	Computerised materials	45 class hours	Present(interactive multiple-choice, short-answer self-assessment test problem solving activities)	High(interactive multiple-choice, short-answer self-assessment test	Present	No delay	Objective(MCQ, short answer question, practical question)	No	No	4
Devitt,2001	Pre-posttest, 2 group; nrss	Australia	Lecture	85/20; Medical students	Ophthalmology	Lecture+e-learning	Medici software	2 weeks	Present(cases)	Low	Absent	No delay	Objective(MCQ)	No	No	5
Mukti,2005	Posttest, 2 groups ; RCT	Malaysia	Traditional collaborative learning	101/85;Undergraduate students	Animal diversity course	lecture + Online collaborative learning	Online web sites	1 semester	Present(group project)	High(collaborative learning, group working)	Present	No delay	Objective(MCQ)	No	No	5
Kiviniemi,2014	Posttest 2-group; NRS	USA	traditional learning	38/28; Public health graduate student	Public health	Online lecture presentation +didactic lecture	web	3 weeks	Absent	High(active learning activity)	Present	No delay	Objective(MCQ, short answer question)	No	No	3
Hsu,2011(a)	Pre-posttest, 2 groups ;NRS	Taiwan	traditional learning	113/88; Nursing students	Nursing ethics	web-based teaching/learning module+ classroom lectures	web-based module(videos, PowerPoint files)	17 weeks	Present(questions and comments)	High(exchange ideas, questions and comments)	Present	No delay	Objective(cannot tell)	No	No	4
Hsu,2011(b)	Pre-posttest, 2 group;	Taiwan	traditional learning	113/88; Nursing students	Nursing ethics	web-based teaching/learning module+ classroom	web-based module(videos, PowerPoint files)	17 weeks	Present(questions and comments)	High(exchange ideas, questions and	Present	No delay	Subjective(cannot tell)	No	No	4

	NRSs					lectures				comments)						
Kaveevivitchai,2009	Pre-posttest, 2 group; RCT s	Thailand	traditional learning	40/40;Nursing students	Anatomy and physiology	CAL multimedia+traditional lecture	interactive CAL multimedia	2 days	Present(questions, case scenarios)	High(questions, case scenarios)	Present	No delay	Objective(MCQ)	No	No	5
Kumrow,2005	Posttest 2-group; NRS	USA	traditional learning	18/15; Graduate nursing students	Health care economic policy and management	Online instruction(50%)+traditional in-classface-to-face(50%)	Web-based	>1 semester	Absent	Low	Absent	No delay	Subjective(self-report)	No	No	3
Howerton,2004(a)	Pre-posttest, 2 groups; RCT	USA	traditional learning	25/24; Dental students	Dental radiology	Interactive CD +lecture	Director 8 authoring software	2 weeks	Present(exercises)	High(exercises, interactive presentation)	Absent	2 weeks	Objective(cannot tell)	No	No	4
Howerton,2004(b)	Pre-posttest, 2 groups; RCT	USA	e-learning	25/26; Dental students	Dental radiology	Interactive CD +lecture	Director 8 authoring software	2 weeks	Present(exercises)	High(exercises, interactive presentation)	Absent	2 weeks	Objective(cannot tell)	No	No	4
Fleetwood,2009	Posttest 2-group; RCT	USA	traditional learning	89/84;Medical students	Bioethics course	Web-based program+lectures +small-group discussions	MedEthEx Online System	8 weeks	Present case, questions with feedback	High(questions with feedback *group discussions)*	Present	3 weeks	Objective(MCQ)	No	No	4
Mars,1996	Pre-posttest, 2 groups ;NRS	Durban	traditional learning	34/34; Medical students	histology	CAI module+	onscreen “patient”	3 weeks	Present(self-assessment questions	High (self-assessment questions ,asking and answering questions)	Absent	No delay	Objective(cannot tell)	No	No	4
Gadbury-Amyot,2012	Posttest 2-group; NRS	USA	traditional learning	309/300; Dental and dental hygiene students	Oral Histology	CAI+lecture	Software standard interactions	>1 semester	Present(questions, self-assessment)	High(interactive multimedia)	Absent	No delay	Objective(cannot tell)	No	No	5
Perkins,2010	Pre-posttest, 2 groups ; RCT	UK	traditional learning	275/276; Medical students	Life support	Face-to-face course +e-learning	Microsim programme on a CD	4 weeks	Absent	Low(Feedback on experiences)	Absent	No delay	Objective(MCQ)	Yes	No	5
Strickland,2008[6][6][26][6]	Pre-posttest, 2 groups	German	traditional learning	8/6; Health professions student	Respiratory Care	Course materials via Internet +face-to-face	Cannot tell	1 semester	Absent	Low	Absent	No delay	Objective(cannot tell)	No	No	3

	;NRS					interaction										
Rouse,2000(a)	Pre-posttest, 2 groups; RCT	USA	traditional learning	20/26; Nursing Students	Pediatric nursing	computer-assisted instruction +traditional class room lecture	CD-ROM, computer	>1 semester	Absent	Low	Absent	No delay	Objective(MCQ)	No	No	5
Rouse,2000(b)	Pre-posttest, 2 groups; RCT	USA	e-learning	20/26; Nursing Students	Pediatric nursing	computer-assisted instruction +traditional class room lecture	CD-ROM, computer	>1 semester	Absent	Low	Absent	No delay	Objective(MCQ)	No	No	5
Gagnon2013	Posttest 2-group; RCT	Canada	traditional learning	52/50; Nursing undergraduates	Critical reading of scientific articles	Internet-based tutorials +in-class sessions;	interactive, Internet-based modules	1 semester	Present(small-group exercises, quizzes)	High(lass discussion, small-group exercises, quizzes.)	Present	No delay	Objective(MCQ, open-ended questions)	No	No	5
Boynton,2007	Posttest 2-group; NRS	USA	traditional learning	98/107; Dental students	Pediatric Behavior Management	Internet-based instructional tool+lectures	web-based instructional tool	6 weeks	Absent	High(essay question)	Present	No delay	Objective(MCQ, short essay)	No	No	3
Lamb,2011	Pre-posttest, 2 groups ;NRS	Uruguay	e-learning	36/30; Health professionals	Tobacco Cessation Skills	Face-toface + online activitie	EviMed system	3 months	Present(cases)	High(cases, wiki-type collaborative activity group-discussion workshops)	Present	No delay	Objective(cannot tell)	No	No	3
Raupach, 2009	Posttest, 2 groups ; RCT	Germany	traditional	72/73; medical students	Clinical reasoning	online module + course	web-based collaborative teaching module	6 weeks	Present(cases)	High(small group discussions)	Present	No delay	Objective(MCQ)	No	No	5
Sherman, 2012	Pre-posttest, 2 groups ; RCT	USA	traditional	35/33;nurses	critical care pharmacology	interactive module+discussion session	interactive learning modules delivered via the hospital's learning management system	1 day	Absent	Low	Present	No delay	Objective(MCQ)	No	No	5
Gerdprasert,2010	Pre-posttest, 2 groups ; RCT	Thailand	traditional	42/43;nursing students	mechanism of labour	web-based learning +conventional lecture	Web-site	2 weeks	Presnt(case scenarios, formative questions and exercises)	High(eb-board for posting questions and discussion between students–students	Present	No delay	Objective(MCQ. True or false question, interctive question)	No	No	5

										and students–teacher)						
Wahlgren,2006	Posttest, 2 groups; RCT	Sweden	traditional	28/85; medical students	dermatology and venereology	conventional teaching +computerised interactsimulation system	computer programming	17 days	Present(cases,quexstions)	High(cases, question, extensive feedback)	Present	No delay	Objective(diagnosis)	No	No	4
**Farrell.2006**	Pre-posttest, 2 groups; RCT	Australia	traditional	35/41; nursing students	pharmacological and clinical contextual knowledge	Mobile Handheld computers+clinical practice	Hewlett Packard PDAs (HP iPAQ Pocket Pc h5500)	3 weeks	Absent	Low	Absent	No delay	Objective(MCQ)	No	No	5
**Taradi,2004**	Posttest, 2 groups; RCT	*Croatia*	traditional	37/84; medical students	acid-base physiology	Online+face-to-face	A Webenvironment created by using the commercially available Web Course Tools (WebCT)	5 weeks	Present(self-testing, exercises, quiz)	High(group collaborations)	Present	No delay	Objective(MCQ, true/false, matching, calculated, short answer, and written paragraph questions)	No	No	4
**Eskenazi, 2010**	Pre-posttest, 2 groups;NRS	Brasil	traditional	41/37;	oral health	Internet-based training p	Cannot tell	3 months	Present(case)	Low(case)	absent	No delay	Objective(cannot tell)	No	No	4

a. no.(B/N) means number of participants in blended learning versus number of participants in no intervention or non-blended learning.

E-Table 4. Quality of included studies

Section 1. Studies comparing blended learning to no intervention

Author, year	Representative intervention group	Comparison group selected from same community	Comparability of cohorts	Blinded outcome assessment	Follow-up adequate	Score
Flys, 2012	Yes	No	Controlled for baseline	Yes	Yes	4
Purl, 2010	Yes	Yes	Controlled for learning outcome and other	Yes	Yes	6
Karaksha, 2011	Yes	Yes	Randomized	Yes	Yes	4
Buchowski, 2002	Yes	No	Controlled for baseline	No	Yes	3
Wallen,2010	Yes	No	Controlled for baseline	No	Yes	3
Weaver,2014(a)	Yes	No	Controlled for baseline	No	Yes	3

Weaver,2014(b)	Yes	No	Controlled for baseline	No	Yes	3
Riesen,2012	Yes	No	Controlled for baseline	No	Yes	3
Cho,2014(a)	Yes	No	Controlled for age	No	Yes	3
Cho,2014(b)	Yes	No	Controlled for age	No	Yes	3
Pereira,2008(a)	Yes	No	Controlled for baseline	Yes	Yes	4
Pereira,2008(b)	Yes	No	Controlled for baseline	Yes	Yes	4
Karamizadeh,2011(a)	Yes	No	Controlled for baseline	No	Yes	3
Karamizadeh,2011(b)	Yes	No	Controlled for baseline	No	Yes	3
Karamizadeh,2011(c)	Yes	No	Controlled for baseline	No	Yes	3
Karamizadeh,2011(d)	Yes	No	Controlled for baseline	No	Yes	3
Karamizadeh,2011(e)	Yes	No	Controlled for baseline	No	Yes	3
Chandler,2008	Yes	No	Controlled for baseline	No	Yes	3
Baumlin 2006	Yes	No	Randomized	No	Yes	3
Cragun, 2005	Yes	No	No	Yes	Yes	3

Section 2.Studies comparing blended learning to non-blended learning

Author, year	Representative intervention group	Comparison group selected from same community	Comparability of cohorts	Blinded outcome assessment	Follow-up adequate	Score
Kulier, 2012	Yes	Yes	Randomized, allocation concealed	Yes	Yes	6
Kavadella, 2012	Yes	Yes	Randomized	No	Yes	4
Lancaster, 2011	Yes	No	Controlled for other	Yes	No	3
Sowan, 2013	Yes	Yes	Randomized, allocation concealed	No	Yes	5
Makhdoom, 2013	Yes	Yes	Randomized	Yes	Yes	5
Lancaster, 2012	Yes	Yes	Randomized	No	Yes	4
Dankbaar, 2014(a)	Yes	Yes	Controlled for age and other	No	Yes	5
Dankbaar, 2014(b)	Yes	Yes	Controlled for age and other	No	Yes	5
Dankbaar, 2014(c)	Yes	Yes	Controlled for age and other	No	Yes	5
Mangione, 1991(a)	Yes	Yes	Randomized	No	Yes	4
Mangione,1991(b)	Yes	Yes	Randomized	No	Yes	4
Stewart, 2013	Yes	Yes	Randomized	No	Yes	4
Mahnken, 2011(a)	Yes	Yes	Randomized	No	Yes	4
Mahnken, 2011(b)	Yes	Yes	Randomized	No	Yes	4
Sung, 2008	Yes	Yes	Controlled for baseline and other	No	Yes	5
Woltering, 2009	Yes	Yes	Controlled for learning and baseline	Yes	Yes	6
Karaksha, 2011(a)	Yes	Yes	Randomized	Yes	Yes	5
Karaksha, 2011(b)	Yes	Yes	Randomized	Yes	Yes	5
Lowe, 2001(a)	Yes	Yes	Controlled for other	Yes	Yes	5
Lowe, 2001(b)	Yes	Yes	Controlled for other	Yes	Yes	5

Ilic, 2013	Yes	Yes	Controlled for other	Yes	Yes	5
Morales, 2012	Yes	Yes	Randomized	Yes	Yes	5
Raupach, 2010	Yes	Yes	Randomized	Yes	Yes	5
Carbonaro, 2008	Yes	Yes	Randomized, allocation concealed	No	Yes	5
Pereira, 2007	Yes	Yes	Controlled for learning and baseline	No	Yes	4
Devitt, 2001	Yes	Yes	Controlled for other	Yes	Yes	5
Mukti, 2005	Yes	Yes	Randomized	Yes	Yes	5
Kiviniemi,2014	Yes	Yes	Controlled for other	No	No	3
Hsu, 2011(a)	Yes	Yes	Controlled for other	No	Yes	4
Hsu, 2011(b)	Yes	Yes	Controlled for other	No	Yes	4
Kaveevivitchai,2009	Yes	Yes	Randomized	Yes	Yes	5
Kumrow,2005	Yes	Yes	No	No	Yes	3
Howerton,2004(a)	Yes	Yes	Randomized	No	Yes	4
Howerton,2004(b)	Yes	Yes	Randomized	No	Yes	4
Fleetwood,2009	Yes	Yes	Randomized	No	Yes	4
Mars,1996	Yes	Yes	Controlled for other	No	Yes	4
Gadbury-Amyot,2012	Yes	Yes	Controlled for age and other	No	Yes	5
Perkins,2010	Yes	Yes	Randomized	Yes	Yes	5
Strickland,2008	Yes	Yes	No	No	Yes	3
Rouse, 2000(a)	Yes	Yes	Randomized	Yes	Yes	5
Rouse, 2000(b)	Yes	Yes	Randomized	Yes	Yes	5
Gagnon,2013	Yes	Yes	Randomized	Yes	Yes	5
Boynton,2007	Yes	Yes	No	No	Yes	3
Lamb,2011	Yes	Yes	No	No	Yes	3
Raupach, 2009	Yes	Yes	Randomized	Yes	Yes	5
Sherman, 2012	Yes	Yes	Randomized	Yes	Yes	5
Gerdprasert,2010	Yes	Yes	Randomized	Yes	Yes	5
Wahlgren,2006	Yes	Yes	Randomized	No	Yes	4
Farrell.2006	Yes	Yes	Randomized	Yes	Yes	5
Taradi,2004	Yes	Yes	Randomized	No	Yes	4
Shomaker, 2002(a)	Yes	Yes	Randomized	Yes	Yes	5
Shomaker, 2002(b)	Yes	Yes	Randomized	Yes	Yes	5
Hilger, 1996	Yes	Yes	Randomized	Yes	Yes	5
Daunt, 2013(a)	Yes	Yes	No	No	Yes	3
Daunt, 2013(b)	Yes	Yes	No	No	Yes	3
Eskenazi, 2010	Yes	Yes	No	No	Yes	3

E-Table 5: GRADE evidence profile

Section 1: Studies comparing blended learning with no intervention

Quality assessment						No of participants		Effect		Quality
No. and design of study	Risk of bias	Inconsistency	Indirectness	Imprecision	Other consideration	X	Y	Relative	Absolut	

					s					
Knowledge score										
2 randomized trials	No serious risk of bias	No serious inconsistency	No serious indirectness	Serious[a]	No	63	67	SMD .59(.001-1.64)	--	⊕⊕⊕○ moderate
18 non-randomized trials	No serious risk of bias	No serious inconsistency	No serious indirectness	No serious imprecision	Large effect size[b]	2006	1861	SMD 1.49(1.11-1.87)	--	⊕⊕⊕○ moderate

a. Sample size is small, and 95%CI is wide.

b. Effect size (1.49) is large.

Section 2. Studies comparing blended learning to non-blended learning

Quality assessment						No of participants		Effect		Quality
No and design of study	Risk of bias	Inconsistency	Indirectness	Imprecision	Other considerations	X	Y	Relative	Absolut	
Knowledge score										
31 randomized trials	Serious[a]	No serious inconsistency	No serious indirectness	No serious imprecision	Reporting bias[b]	1358	1359	SMD . 75(.38-1.12)	--	⊕⊕○○ low
25 non-randomized trials	No serious risk of bias	No serious inconsistency	No serious indirectness	No serious imprecision	Large effect size[c], reporting bias[2]	1451	1270	SMD .87(.56-1.05)	--	⊕⊕○○ low

a. Allocation concealed was not described in 28 studies.
b. Reporting bias was found.
c. The effect size .87(.56-1.05) was large.

E-Table 6. Standard knowledge score and source

Section 1. Studies comparing blended learning to no intervention

	Intervention			Control			Source
	**No.**	**Standard Mean**	**Standard SD**	**No.**	**Standard Mean**	**Standard SD**	

Flys, 2012	225	90.3	10.71	225	70.9	16.84	Mean,95%CI
Purl, 2010	350	44.33	1.07	102	41.87	1.93	Mean, SD
Karaksha, 2011	23	66.96	23.82	17	45.88	18.39	Mean, SD
Buchowski, 2002	14	73	12	14	31	7	Mean, SD
Wallen,2010	16	56.39	20.02	16	37.09	16	Mean, SD
Weaver,2014(a)	80	76.81	13.43	78	74.13	13.33	Mean, average SD
Weaver,2014(b)	58	74.45	13.43	127	76.03	13.33	Mean, average SD
Riesen,2012	60	84.43	6.54	60	82.1	6.96	Mean, SD
Cho,2014(a)	60	79.2	16.6	60	64.8	13.4	Mean, SD
Cho,2014(b)	60	77.2	12	60	53.4	10	Mean, SD
Pereira,2008(a)	69	78	9.5	69	60.5	13.2	Mean, SD
Pereira,2008(b)	45	80	1.25	45	46.25	15	Mean, SD
Karamizadeh,2011(a)	6	100	11	6	80	11	Mean, SD
Karamizadeh,2011(b)	38	69	24	38	49	19	Mean, SD
Karamizadeh,2011(c)	19	85	22	19	44	15	Mean, SD
Karamizadeh,2011(d)	40	86	16	40	42	19	Mean, SD
Karamizadeh,2011(e)	10	70	31	10	42	14	Mean, SD
Chandler,2008	817	94.25	8.07	817	72.17	16.31	Mean, SD
Baumlin 2006	40	72.8	13.43	50	68.2	13.33	Mean, average SD
Cragun, 2005	39	74	13.5	15	62	13.5	Mean, SD

Section 2. Studies comparing blended learning to non-blended learning

	Intervention			Control			Source
	**No.**	**Standard Mean**	**Standard SD**	**No.**	**Standard Mean**	**Standard SD**	
Kulier, 2012	123	69.52	5.95	81	61.45	6.20	Mean,95%CI
Kavadella, 2012	24	80.88	13.82	22	68.64	13.90	Mean, SD
Lancaster, 2011	97	84.09	8.98	97	65.15	10.14	Mean, average SD
Sowan, 2013	105	78.00	5.50	105	70.00	8.50	Mean, SD
Makhdoom, 2013	60	71.69	12.31	61	66.02	11.82	Mean, SD
Lancaster,2012	29	96.60	1.90	23	92.70	3.80	Mean, SD
Dankbaar, 2014(a)	31	80.00	2.00	16	80.00	3.00	Mean, SD

Dankbaar, 2014(b)	31	76.00	2.00	16	75.00	3.00	Mean, SD
Dankbaar, 2014	31	73.00	2.00	16	68.00	3.00	Mean, SD
Mangione, 1991(a)	13	78.50	18.28	13	70.00	22.28	Mean, SD
Mangione, 1991(b)	13	78.50	18.28	9	62.50	19.85	Mean, SD
Shomaker, 2002(a)	24	44.60	8.98	24	51.00	10.14	Mean, average SD
Shomaker, 2002(b)	24	44.60	8.98	17	51.20	10.14	Mean, average SD
Stewart, 2013	34	75.00	12.25	37	67.50	11.75	Mean, SD
Mahnken, 2011(a)	32	72.90	12.30	32	69.00	12.40	Mean, SD
Mahnken, 2011(b)	32	87.70	12.80	32	69.00	12.40	Mean, SD
Sung, 2008	24	82.21	8.75	26	67.92	7.17	Mean, SD
Woltering, 2009	74	63.20	14.08	71	55.76	12.28	Mean, SD
Karaksha, 2011(a)	23	66.96	23.82	13	54.55	26.32	Mean, SD
Karaksha, 2011(b)	23	66.96	23.82	22	41.54	22.30	Mean, SD
2e, 2001(a)	39	31.80	15.20	46	25.00	16.70	Mean, SD
2e, 2001(b)	39	50.30	14.00	46	50.20	17.40	Mean, SD
Hilger, 1996	45	78.40	8.98	32	73.40	10.14	Mean, average SD
Ilic, 2013	34	40.53	18.00	27	45.13	22.40	Mean, SD
Daunt, 2013(a)	92	92.00	8.98	67	85.10	10.14	Mean, average SD
Daunt, 2013(b)	162	84.20	8.98	168	68.40	10.14	Mean, average SD
Morales, 2012	22	72.30	6.20	22	74.20	8.10	Mean, SD
Raupach, 2010	40	84.80	1.30	34	79.50	1.40	Mean, SD
Carbonaro, 2008	22	32.44	7.33	22	34.00	10.67	Mean, SD
Pereira, 2007	65	63.00	13.00	65	50.00	16.00	Mean, SD
Devitt, 2001	85	61.67	1.11	20	45.00	2.50	Mean, SD
Mukti, 2005	101	61.77	9.98	85	45.38	11.66	Mean, SD
Kiviniemi,2014	38	93.92	2.45	28	91.76	4.95	Mean, SD
Hsu, 2011(a)	113	80.28	10.84	88	81.96	10.56	Mean, SD
Hsu, 2011(b)	113	66.41	8.46	88	68.11	8.73	Mean, SD
Kaveevivitchai, 2009	40	61.10	6.23	40	59.43	7.83	Mean, SD
Kumrow, 2005	18	97.15	2.56	15	94.78	3.37	Mean, SD
Howerton, 2004(a)	25	84.40	9.28	24	82.50	12.07	Mean, SD

Howerton, 2004(b)	25	84.40	9.28	26	75.00	7.07	Mean, SD
Fleetwood, 2009	89	83.00	5.00	84	83.00	5.00	Mean, SD
Mars, 1996	34	65.60	8.98	34	60.70	10.14	Mean, average SD
Gadbury-Amyot, 2012	309	95.75	10.00	300	92.00	12.75	Mean, SD
Perkins, 2010	275	84.50	11.58	276	84.92	11.50	Mean, SD
Strickland, 2008	8	86.00	8.98	6	85.00	10.14	Mean, average SD
Rouse, 2000(a)	20	77.30	11.50	26	66.20	11.60	Mean, SD
Rouse, 2000(b)	20	77.30	11.50	26	74.00	11.00	Mean, SD
Gagnon, 2013	52	17.20	0.90	50	14.50	0.60	Mean, SD
Boynton, 2007	98	78.22	7.67	107	74.72	12.56	Mean, SD
Lamb, 2011	36	83.10	2.80	30	75.30	17.20	Mean, SD
Raupach, 2009	73	74.00	10.00	72	74.00	9.60	Mean, SD
Sherman, 2012	35	89.7	5.16	33	88.30	6.79	Mean, SD
Gerdprasert, 2010	42	71.90	9.59	43	87.93	5.76	Mean, SD
Wahlgren, 2006	28	88.80	9.38	85	87.50	10.00	Mean, SD
Farrell, 2006	35	50.66	8.98	41	45.34	10.14	Mean, average SD
Taradi, 2004	37	71.69	1.83	84	61.33	1.03	Mean, SD
Eskenazi, 2010	41	33.30	8.98	37	30.30	10.14	Mean, average SD

E-table 7. PRISMA 2009 Checklist

**Section/topic**	**#**	**Checklist item**	**Reported on heading**
**TITLE**			
on	1	Antenatal depressive symptoms and the risk of preeclampsia or operative deliveries: A meta-analysis	Title (page 1)
**ABSTRACT**			
Structured summary	2	Provide a structured summary including, as applicable: background; objectives; data sources; study eligibility criteria, participants, and interventions; study appraisal and synthesis methods; results; limitations; conclusions and implications of key findings; systematic review registration number.	Abstract (page 2-3)
**INTRODUCTION**			

Rationale	3	Describe the rationale for the review in the context of what is already known.	Introduction (page 4)
Objectives	4	Provide an explicit statement of questions being addressed with reference to participants, interventions, comparisons, outcomes, and study design (PICOS).	Introduction (page 4-5)
**METHODS**			
Protocol and registration	5	Indicate if a review protocol exists, if and where it can be accessed (e.g., Web address), and, if available, provide registration information including registration number.	N/A
Eligibility criteria	6	Specify study characteristics (e.g., PICOS, length of follow-up) and report characteristics (e.g., years considered, language, publication status) used as criteria for eligibility, giving rationale.	Methods: Eligibility criteria (page 5-6)
Information sources	7	Describe all information sources (e.g., databases with dates of coverage, contact with study authors to identify additional studies) in the search and date last searched.	Methods: Data sources (page 6)
Search	8	Present full electronic search strategy for at least one database, including any limits used, such that it could be repeated.	Methods: e-table 1 (supplemental document)
Study selection	9	State the process for selecting studies (i.e., screening, eligibility, included in systematic review, and, if applicable, included in the meta-analysis).	Methods: Study selection (page 6-7)
Data collection process	10	Describe method of data extraction from reports (e.g., piloted forms, independently, in duplicate) and any processes for obtaining and confirming data from investigators.	Methods: Data extraction (page 7)

Section/topic	#	Checklist item	Reported on Heading
**METHODS (cont.)**			
Data items	11	List and define all variables for which data were sought (e.g., PICOS, funding sources) and any assumptions and simplifications made.	Methods: Data extraction (page 7)
Risk of bias in individual studies	12	Describe methods used for assessing risk of bias of individual studies (including specification of whether this was done at the study or outcome level), and how this information is to be used in any data synthesis.	Methods: Quality Assessment(page 7-8)

Summary measures	13	State the principal summary measures (e.g., risk ratio, difference in means).	Methods: Data Synthesis(page 9)
Synthesis of results	14	Describe the methods of handling data and combining results of studies, if done, including measures of consistency (e.g., I^2) for each meta-analysis.	Methods: Data Synthesis(page 9)
Risk of bias across studies	15	Specify any assessment of risk of bias that may affect the cumulative evidence (e.g., publication bias, selective reporting within studies).	Methods: Data Synthesis(page 9
Additional analyses	16	Describe methods of additional analyses (e.g., sensitivity or subgroup analyses, meta-regression), if done, indicating which were pre-specified.)	Methods: Data Synthesis(page 9)
**RESULTS**			
Study selection	17	Give numbers of studies screened, assessed for eligibility, and included in the review, with reasons for exclusions at each stage, ideally with a flow diagram.	Results: Study selection and Figure 1 (page 9-10)
Study characteristics	18	For each study, present characteristics for which data were extracted (e.g., study size, PICOS, follow-up period) and provide the citations.	Results: Table 1 (page 11-12) and e-table 3 (supplemental document)
Risk of bias within studies	19	Present data on risk of bias of each study and, if available, any outcome level assessment (see item 12).	Results: Study quality(page 12-13)
Results of individual studies	20	For all outcomes considered (benefits or harms), present, for each study: (a) simple summary data for each intervention group (b) effect estimates and confidence intervals, ideally with a forest plot.	Results: Figure 2 (page 14) and Figure 4 (page 18),

			and e-table 6 (supplemental document)
Synthesis of results	21	Present results of each meta-analysis done, including confidence intervals and measures of consistency.	Results: Figure 2 (page 14) and Figure 4 (page 18)
**Section/topic**	**#**	**Checklist item**	**Reported on Heading**
**RESULTS (cont.)**			
Risk of bias across studies	22	Present results of any assessment of risk of bias across studies (see Item 15).	Results: Figure 3(page 15) and Figure 5 (page 19)
Additional analysis	23	Give results of additional analyses, if done (e.g., sensitivity or subgroup analyses, meta-regression [see Item 16]).	Results: Table 2 (page 16-17)and Table 3 (page 20-21)
**DISCUSSION**			
Summary of evidence	24	Summarize the main findings including the strength of evidence for each main outcome; consider their relevance to key groups (e.g., healthcare providers, users, and policy makers).	Discussion (page 22-24)
Limitations	25	Discuss limitations at study and outcome level (e.g., risk of bias), and at review-level (e.g., incomplete retrieval of identified research, reporting bias).	Limitations and strengths (page 24-25)
Conclusions	26	Provide a general interpretation of the results in the context of other evidence, and implications for future research.	Conclusion (page 26-27)
**FUNDING**			
Funding	27	Describe sources of funding for the systematic review and other support (e.g., supply of data); role of funders for the systematic review.	Acknowledgements (page 27)

From: Moher D, Liberati A, Tetzlaff J, Altman DG, The PRISMA Group (2009). Preferred Reporting Items for Systematic Reviews and Meta-Analyses: The PRISMA Statement. PLoS Med 6(6): e1000097. doi:10.1371/journal.pmed1000097
